# Protective Effects of Cannabidiol on the Membrane Proteome of UVB-Irradiated Keratinocytes

**DOI:** 10.3390/antiox10030402

**Published:** 2021-03-08

**Authors:** Sinemyiz Atalay, Agnieszka Gęgotek, Elżbieta Skrzydlewska

**Affiliations:** Department of Analytical Chemistry, Medical University of Białystok, 15-089 Białystok, Poland; sinemyiz.atalay@umb.edu.pl (S.A.); agnieszka.gegotek@umb.edu.pl (A.G.)

**Keywords:** keratinocytes, UVB radiation, cannabidiol, membrane proteins, oxidative stress, redox balance, protein degradation, apoptosis, adducts of protein-lipid peroxidation products, proteomic analysis

## Abstract

Ultraviolet (UV) radiation contained in sunlight disturbs the redox state of skin cells, leading to changes in the structures and functions of macromolecules including components of biological membranes. Cannabidiol (CBD), which accumulates in biomembranes, may be a promising protective antioxidant compound. Accordingly, the aim of this study was to compare the effects of short-term (24 h) and long-term (48 h) CBD application on the proteomic profile of biological membranes in UVB-irradiated keratinocytes. The data obtained show that UVB radiation quantitatively and qualitatively modified cell membrane proteins, with a particular research focus on adducts of proteins with the lipid peroxidation products malondialdehyde (MDA) or 4-hydroxynonenal (4-HNE). CBD application reduced the UVB-enhanced level of these protein adducts. This was particularly notable amongst proteins related to cell proliferation and apoptosis. Moreover, CBD dramatically increased the UVB-induced expression of proteins involved in the regulation of protein translation and cell proliferation (S3a/L13a/L7a ribosomal proteins), the inflammatory response (S100/S100-A6 proteins), and maintenance of redox balance (peroxiredoxin-1, carbonyl reductase 1, and aldo-keto reductase family 1 members). In contrast, CBD effects on the level of 4-HNE-protein adducts involved in the antioxidant response and proteasomal degradation process indicate that CBD may protect keratinocytes in connection with protein catabolism processes or pro-apoptotic action.

## 1. Introduction

Ultraviolet (UV) radiation, as a component of solar radiation to which people are exposed daily, causes redox imbalance and inflammation, which may play a role in initiating chronic skin diseases [1,2,3]. The cells of the epidermis including the basic building blocks such as keratinocytes are particularly exposed to UV radiation [4]. UVB radiation (290–320 nm), which is absorbed mainly by the epidermis, inhibiting apoptosis and enhancing the proliferation of epidermal cells, can intensify mutagenesis, causing skin cancer [5]. UVB radiation may disturb the integrity of the genome, causing various DNA mutations [6] as well as enhancing the biosynthesis of cytokines and vasoactive and neuroactive mediators, causing an inflammatory reaction [2]. In addition, UVB, by changing the metabolism and physiology of keratinocytes, can induce apoptosis by activating the p53 protein [7]. However, after prolonged exposure, keratinocytes, influenced by various growth factors, may begin to multiply strongly in order to protect the skin from deep radiation penetration [8]. By initiating the inflammatory process, UVB promotes increased generation of ROS and the reduction of the antioxidant capacity of cells, which results in oxidative stress and damage to biologically important macromolecules including DNA, lipids, and proteins [9]. Moreover, it has been shown in a model corresponding to the human epidermis that UVB can significantly increase the activity of glucose-6-phosphate dehydrogenase, which initiates glucose catabolism, especially in cells requiring DNA repair [10]. The metabolic consequences caused by UV radiation necessitate the search for protective and/or therapeutic compounds to prevent skin diseases.

One of the increasingly interesting natural compounds with antioxidant and anti-inflammatory properties is cannabidiol (CBD), a non-psychoactive phytocannabinoid found in *Cannabis sativa* L. [11]. Like endogenous cannabinoids, the biological activity of CBD may manifest through the regulation/activation of membrane receptors, which participate in the regulation of the level of ROS and pro-inflammatory interleukins [12]. In addition, a recent study using an aqueous extract obtained from a cannabis strain that does not produce THC, but contains CBD and other phenolic compounds, showed that this extract restores hydrogen peroxide–reduced gene expression of interleukin-6, which is a key pro-inflammatory cytokine involved in the differentiation, activation, and proliferation of skin keratinocytes [13]. CBD has been shown to modulate the antioxidant capacity of cells by inducing the transcription of cytoprotective compounds including antioxidants by the nuclear factor associated with erythroid 2 (Nrf2) and inhibiting the transcription of nuclear factor kappa B (NF-κB) [12,14]. This situation leads to intensification of antioxidant potential and limits oxidative modification of cell components including biological membranes, which is confirmed, inter alia, by the increased integrity of cell membranes, as demonstrated by their reduced permeability [15].

Changes to membrane structure and/or signaling pathways caused by such modifications are important to the protective effect of CBD. Recently, it has been shown that CBD’s effect on keratinocyte metabolism including redox balance and the oxidative stress resulting from its disturbance depends on the way in which CBD is administered (i.e., before and after UVB irradiation vs. afterward only) [15]. The mechanism leading to this variation can be observed in the significantly lower levels of CBD in the cytosol than in membranes, especially in keratinocytes subjected to long-term CBD therapy (i.e., before and after UVB irradiation).

Based on the above, the aim of this study was to assess the effect/not of CBD on the proteomic profile of cell membranes in vitro when administered in either of two ways: (1) before and after irradiation of keratinocytes with UVB radiation, or (2) only after irradiation.

## 2. Materials and Methods

### 2.1. Cell Culture and Experimental Cell Groups

Human keratinocytes (CDD 1102 KERTr) obtained from American Type Culture Collection (ATCC, Virginia, USA) were cultured in Dulbecco’s modified Eagle’s medium (DMEM), together with 10% fetal bovine serum (FBS) supplemented with 50 U/mL penicillin and 50 μg/mL streptomycin. Cells were cultured in a humidified atmosphere of 5% CO_2_ at 37 °C. After keratinocytes were grown to 70% confluence, they were passaged at a ratio of 1:3. After reaching the required confluency for all passages, keratinocytes were divided into two main cell groups as described below: 

Control groups of keratinocytes

- Control cells cultured with standard medium (CTR).
- Cells cultured in medium containing 4 µM CBD (Sigma-Aldrich, MO, USA—98.5% pure) in 0.2% ethanol for 48 h (CBD(48 h))
- Cells cultured in medium containing 4 µM CBD in 0.2% ethanol for 24 h (CBD(24 h)).

UVB-irradiated groups of keratinocytes

IV. Cells irradiated by UVB (312 nm) at 60 mJ/cm^2^ (Bio-Link Crosslinker BLX 312; Vilber Lourmat, Germany; six lamps at a distance of 15 cm) (UVB).
V. Cells cultured in medium containing 4 μM CBD in 0.2% ethanol for 24 h before and 24 h after UVB irradiation (pretreatment + treatment) (CBD + UVB + CBD)
VI. Cells cultured in medium containing 4 μM CBD in 0.2% ethanol for 24 h after UVB irradiation (treatment only) (UVB + CBD).

### 2.2. Cell Membrane Fractionation

After completion of the experiment at the cell culture level, cells were obtained by centrifugation (300× *g*, 3 min) and re-suspended in Tris-buffered saline (TBS) supplemented with a mixture of proteasome inhibitors (pH 8.0) before being centrifuged (15,000× *g*, 10 min). After centrifuging, the pellet was re-suspended in TBS supplemented with 1% Triton X-100 and sonicated. After sonication, samples were centrifuged (15,000× *g*, 10 min). Next, membrane fractions were harvested for use in downstream proteomic analysis (see below). The total protein content of the samples was measured by the Bradford assay [16].

### 2.3. Proteomic Analysis

Samples containing 30 µg of protein together with loading buffer (Laemmli buffer containing 5% 2-mercaptoethanol) at a volume ratio of 1:2 were heated at 100 °C for 7 min, then separated using 10% Tris-Glycine sodium dodecyl sulfate-polyacrylamide gel electrophoresis (SDS-PAGE) gels. Following electrophoretic protein separation, gels were fixed in a 4:1:5 mixture of methanol:acetic acid:water for 1 h and stained overnight with Coomassie Brilliant Blue R-250. The lines were cut from the gels and sliced into eight sections. The proteins in each slice were reduced using 10 mm dithiothreitol (DTT) and alkylated with 50 mm iodoacetamide. Overnight in-gel protein digestion was then carried out using trypsin (Promega, Madison, WI, USA). The resulting peptide mixtures were extracted from the gel and dried. Dried peptide mixtures were dissolved in 5% acetonitrile with 0.1% formic acid, then these final peptide mixtures were captured on a 300 µm inside diameter × 5 mm long C18 μ-Precolumn (Dionex, LC Packings) and then loaded onto a 150 mm × 75 mm PepMap RSLC capillary analytical C18 column with 2 µm particle size (Dionex, LC Packings) at a flow rate of 0.3 µL/min using an Ultimate 3000 (Dionex, Idstein, Germany) for separation. Samples were mobilized through the column by application of two eluents over a time gradient. Eluent A consisted of 5% acetonitrile + 0.1% formic acid. The gradient to eluent B, consisting of 90% acetonitrile + 0.03% formic acid was initiated after 3 min, rising over the subsequent 40 min to a final ratio of 40% eluent A/60% eluent B. Eluted peptides were analyzed using a Q Exactive HF mass spectrometer with an electrospray ionization source (ESI) (Thermo Fisher Scientific, Bremen, Germany). The mass spectrometer was calibrated and operated in positive and data-dependent mode. Data were acquired using Xcalibur software (Thermo Fisher Scientific, Bremen, Germany). Peptide identification was conducted by liquid chromatography-tandem mass spectrometry (LC-MS/MS) according to the technique described previously by Gęgotek [17].

### 2.4. Identification and Quantification of Proteins

Raw data generated by LC-MS/MS were analyzed using Proteome Discoverer 2.0 (Thermo Fisher Scientific, Bremen, Germany) and MS Amanda (MS Amanda algorithm, license Thermo Scientific, a registered trademark of the University of Washington, Seattle, WA, USA). In the process of protein identification, the following search parameters were used: peptide mass tolerance 10 ppm; MS/MS mass tolerance 0.02 Da; mass precision 2 ppm; up to two missed cleavages allowed; minimal peptide length of six amino acids; and cysteine carbamidomethylation and carboxymethylation, methionine oxidation, MDA-lysine, and 4-HNE-cysteine/lysine/histidine adduct formation set as a dynamic modification. For each protein, the minimum number of identified unique peptides was set to two. Input data were searched against the UniProtKB-SwissProt database (taxonomy: *Homo sapiens*, release July 2020). In accordance with the signal intensities of the precursor ions, protein label-free quantification (LFQ) was performed.

### 2.5. Statistical Analysis

**Figure 1:**
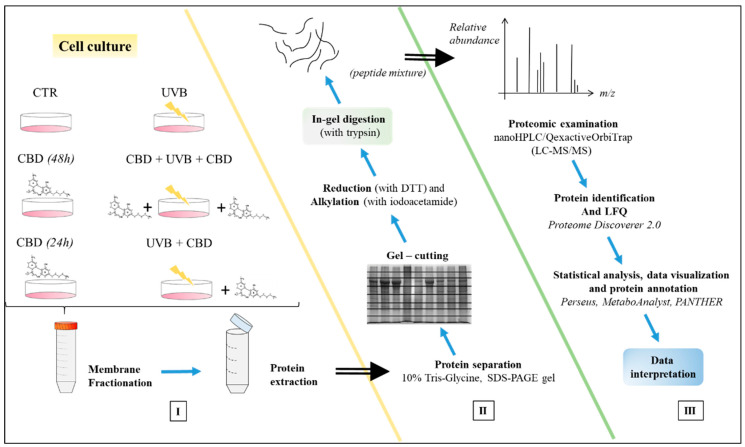
The main stages of proteomic analysis of keratinocyte membranes: **I.** Preparation of keratinocytes (six groups as described above) for obtaining cell membranes and extraction membrane proteins; **II.** Preparation of proteins for proteomic analysis; **III.** Proteomic qualitative–quantitative analysis.

Samples from each experimental cell group were analyzed in three independent experiments. The effects of CBD pre-treatment (CBD + UVB + CBD) and CBD treatment (UVB + CBD) on membrane obtained keratinocytes exposed to UVB were analyzed separately. The results from individual protein label-free quantification were normalized by Z-score and log-transformed using open-source software Perseus (Perseus 1.6.5.0, https://maxquant.net/perseus/ (accessed on 18 September 2020) [18]. Quality control and biostatistical analysis including one-way ANOVA (analysis of variance), post-hoc test (controlling the false discovery rate–FDR, Benjamini–Hochberg adjustment), principal component analysis (PCA), volcano plots, and heat map were completed using Perseus and open-source software MetaboAnalyst 4.0 (http://www.metaboanalyst.ca (accessed 21 September 2020) [19]. Protein annotations were conducted using open-source software PANTHER (http://pantherdb.org/ (accessed on 23 September 2020) [20] and compared against The National Center for Biotechnology Information (NCBI) database (https://www.ncbi.nlm.nih.gov/ (accessed on 28 September 2020) and the open-source database STRING v11 (https://string-db.org/ (accessed on 29 September 2020) [21]. Additionally, for descriptive analysis of proteins found to participate in signaling in critical biological pathways (apoptosis, antioxidant defense, proteasomal degradation, calcium dependent protein binding) and whose expression changes significantly (differences were considered significant when *p* ≤ 0.05), boxplots were generated using Excel (for the quartile calculation, inclusive median was used). The main stages of all proteomic analysis are shown in Figure 1.

## 3. Results

**Figure 2:**
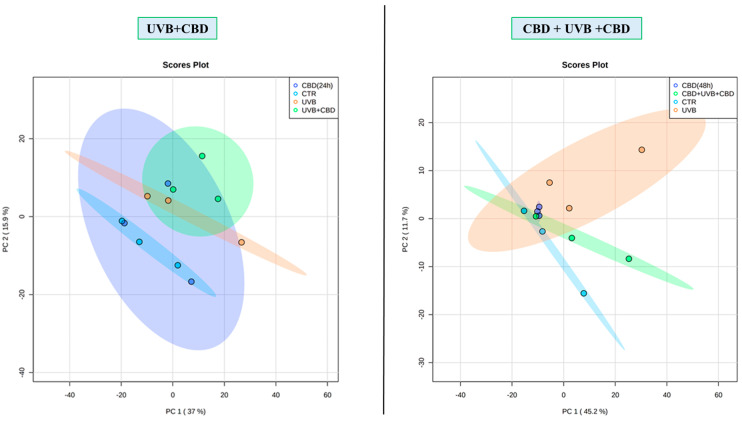
Principal component analysis (PCA) of membrane fractions proteins of keratinocytes from the following groups: CTR—control cells cultured with standard medium; CBD(48 h)—cells cultured in medium containing CBD (4 µM) for 48 h; CBD(24 h)—cells cultured in medium containing CBD (4 µM) for 24 h; UVB—cells irradiated with UVB (312 nm) at 60 mJ/cm^2^; CBD + UVB + CBD—cells cultured in medium containing CBD (4 µM) for 24 h before and after UVB irradiation; UVB + CBD—cells cultured in medium containing CBD (4 µM) for 24 h after UVB irradiation.

**Figure 3:**
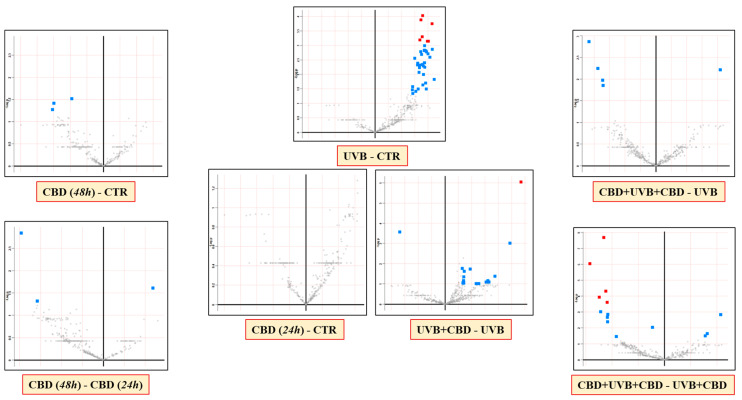
Volcano plots comparing the effects of cannabidiol (CBD) on membrane fractions proteins of keratinocytes from the following groups: CTR—control cells cultured with standard medium; CBD(48 h)—cells cultured in medium containing CBD (4 µM) for 48 h; CBD(24 h)—cells cultured in medium containing CBD (4 µM) for 24 h; UVB—cells irradiated with UVB (312 nm) at 60 mJ/cm^2^; CBD + UVB + CBD—cells cultured in medium containing CBD (4 µM) for 24 h before and after UVB irradiation; and UVB + CBD—cells cultured in medium containing CBD (4 µM) for 24 h after UVB irradiation. [significant features (in blue) had *p* < 0.05; significant features (in red) had FDR-adjusted *p*-value < 0.05].

In this study, a total of 512 proteins was identified at a detection sensitivity of at least two unique peptides, and their levels were identified in the membrane fractions of all experimental groups. The list of identified proteins, their names, related assigned peptide numbers, sequence coverages as well as their average levels in each group is presented in Table S1. The PCA demonstrated that long-term application of CBD favored more clustering of experimental groups within themselves, with increasing variation in protein expression between cell groups (CBD(48 h)/CBD + UVB + CBD; Component 1–45.2%; Component 2–11.7%; Figure 2). However, in the case of shorter-term application of CBD, all cell groups showed a clustering closer to each other (CBD(24 h)/UVB + CBD; Component 1–37 %; Component 2–15.9%). It was also observed that the CBD(48 h) group clustered more closely to the CTR group than to the CBD(24 h) group. On the other hand, volcano plots comparing the CTR and UVB groups with the groups treated with CBD (CBD(24 h) and CBD(48 h)) showed that UVB irradiation causes a remarkably significant differences in membrane proteome compared to the control cells (CTR), while the application of CBD caused negligible (CBD(48 h)) or no difference (CBD(24 h)) (Figure 3). Moreover, volcano plots comparing CBD treated cell groups (CBD + UVB + CBD and UVB + CBD) with UVB groups showed that the membrane proteomes of UVB-irradiated keratinocytes differed significantly for the two different CBD application times.

**Figure 4:**
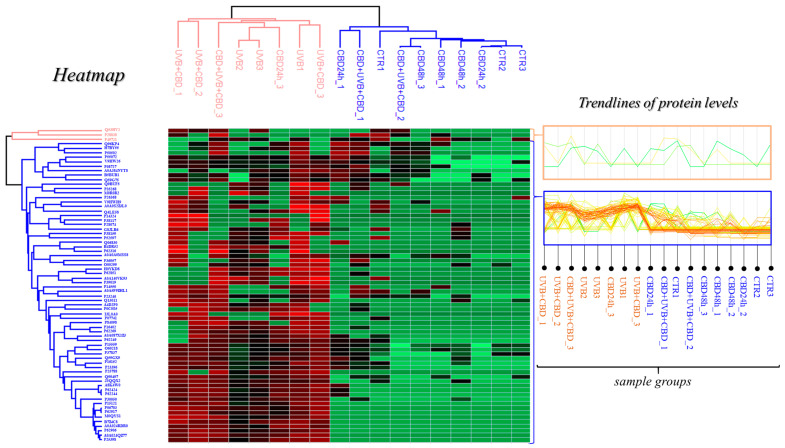
Heat map and clustering for significant proteins from the following groups: CTR—control cells cultured with standard medium (*n* = 3); CBD(48 h)—cells cultured in medium containing CBD (4 µM) for 48 h (*n* = 3); CBD(24 h)—cells cultured in medium containing CBD (4 µM) for 24 h (n = 3); UVB—cells irradiated with UVB (312 nm) at 60 mJ/cm^2^ (*n* = 3); CBD + UVB + CBD—cells cultured in medium containing CBD (4 µM) for 24 h before and after UVB irradiation (*n* = 3); UVB + CBD—cells cultured in medium containing CBD (4 µM) for 24 h after UVB irradiation (*n* = 3).

Statistical comparison of all cell groups showed 70 proteins with significantly altered expression. These are primarily ribosomal, and are involved in the processes of translation, ubiquitination of proteins, and cell signaling as well as proteasomal, catalytic, DNA/RNA binding, and structural proteins (Table S2). Proteins and their cellular locations under physiological conditions (cytosol, plasma membrane, extracellular exosome, lysosome, mitochondria, Golgi apparatus, endoplasmic reticulum, nucleus) are shown with their fold-changes between cell groups: UVB vs. CTR; CBD(48 h)/CBD(24 h) vs. CTR; CBD + UVB + CBD vs. UVB; and UVB + CBD vs. UVB (Table S2). The overall picture from the heat map and Table S2 showed that UVB irradiation caused a significant increase in the expression levels of almost all 70 proteins, the levels of which changed significantly between cell groups (Figure 4, Table S2). At the same time, it was found that the short-term effect of CBD (UVB + CBD) further enhanced this increase in expression; however, long-term CBD treatment (CBD + UVB + CBD) showed a tendency to compensate for this increased growth (Figure 4, Table S2). On the other hand, when considering the heat map, the primary split in the upper hierarchical dendrogram showed that samples from the groups CBD(48 h) and CBD + UVB + CBD clustered closer, independently from the samples exposed to UVB radiation (Figure 4). Even if the results from the CTR group were not completely clustered, they were separate from the groups of cells exposed to UVB, and clustered more closely to the groups of cells treated with CBD (CBD(48 h) and CBD(24 h)). The heat map clusters indicate that the UVB + CBD group was clustered closer to the UVB group, while the CBD + UVB + CBD group was almost entirely clustered outside the UVB group. Thus, both UVB radiation and the use of CBD (CBD + UVB + CBD or UVB + CBD) significantly changed the proteomic profile of keratinocyte membranes. However, the use of CBD in the CBD(48 h) or CBD(24 h) regimes did not cause significant differences in the membrane proteome compared to the CTR group.

A comparison of proteins whose levels changed statistically between groups showed many changes in the ribosomal protein family (Table S2). UVB radiation remarkably enhanced the level of all identified ribosomal proteins (mainly related to membranes: nuclear, cytosolic or extracellular exosome) including: 40S RPs (S15a (P62244), S19 (P39019), S3 (P23396), S3a (A8K4W0), S25 (P62851)); 60S RPs (L7a (P62424), L13a (M0QYS1), L31(B7Z4C8), L22 (P35268)); ribosomal protein L5 variant (Q59GX9); and eIF4G1 variant protein (Q4LE58). Additionally, treatment with CBD for 24 h generally increased the levels of these proteins, unlike CBD treatment for 48 h, which did not cause significant differences in the levels of most of these proteins compared to the CTR group. CBD + UVB treatment additionally increased the UVB-induced level of these proteins, but CBD + UVB + CBD treatment caused a lesser increase (or even decrease) in the proteins listed above (with the exception of S15a) (Table S2). The level of transitional endoplasmic reticulum ATPase (P55072) increased after UVB irradiation, but CBD treatment (both CBD + UVB and CBD + UVB + CBD) lowered its levels. For proteins of membrane fractions, mainly elevated levels were observed in the UVB + CBD group, with decreased (or less elevated) levels in the CBD + UVB + CBD group. This also applied to DNA/RNA binding proteins such as RNA-binding motif protein (P381590), histone H2B (B4DR52), and histone H2A.Z (P0C0S5).

**Figure 5:**
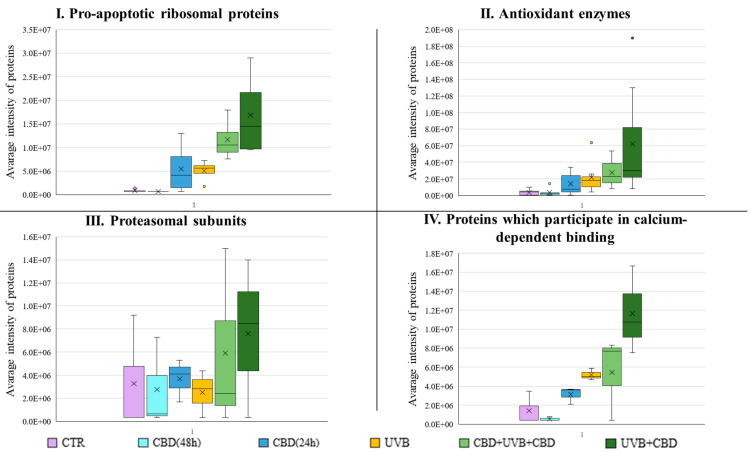
The box plots generated using the average intensities of the proteins in the specified protein groups: (**I**) Pro-apoptotic ribosomal proteins: ribosomal proteins S3, S19, L13a, L7a; (**II**) Antioxidant enzymes: aldo-keto reductase family 1 member A1, B1, B10, C3, carbonyl reductase [NADPH] 1, NAD(P)H dehydrogenase [quinone] 1, peroxiredoxin-1; (**III**) Proteasomal subunits: proteasome subunit alpha type-3, beta type-2, beta type-5; and (**IV**) Proteins that participate in calcium-dependent binding: EF-hand domain family-member D2, protein S100 and S100-A6. [CTR—control cells cultured with standard medium (*n* = 3); CBD(48 h)—cells cultured in medium containing CBD(4 µM) for 48 h (*n* = 3); CBD(24 h) – cells cultured in medium containing CBD(4 µM) for 24 h (*n* = 3); UVB—cells irradiated with UVB (312 nm) at 60 mJ/cm^2^ (*n* = 3); CBD+UVB+CBD—cells cultured in medium containing CBD(4 µM) for 24 h before and after UVB irradiation (*n* = 3); UVB+CBD—cells cultured in medium containing CBD(4 µM) for 24 h after UVB irradiation (*n* = 3)].

According to the box plots (Figure 5), the intra-group variations in protein expression from the CTR and CBD(48 h) groups were small (for protein groups indicated in the figure: pro-apoptotic ribosomal proteins, antioxidant enzymes). In contrast, the intra-group variability of these proteins was extremely high in groups UVB + CBD, CBD + UVB + CBD, and CBD(24 h). Additionally, in the group of proteins participating in calcium-dependent binding, high variability was found in the UVB + CBD and CBD + UVB + CBD groups. Proteasomal subunits showed the highest intra-group variations for all keratinocyte groups. Comparison of medians indicated that variation between the CTR and CBD(48 h) groups was negligibly less in the protein group of pro-apoptotic ribosomal proteins and antioxidant enzymes. Furthermore, in the same protein groups, there was inter-group variation between both of the CTR and CBD(48 h) groups and other cell groups. Particularly in the group of pro-apoptotic ribosomal proteins, there was an obvious significant variation between the groups CBD + UVB + CBD, UVB + CBD, and UVB alone.

Detailed analysis showed that UVB radiation increased the levels of HCG2044781 (I3L0A0), proteasome activator complex subunit 2 (A0A087X1Z3), proteasome subunit alpha type-3 (P25788), and the Hsc70-interacting protein (P50502). All CBD + UVB + CBD and UVB + CBD groups had elevated levels of proteasome activator complex subunits 2 and alpha type-3 relative to the UVB-only group, while levels of HCG2044781 and Hsc70 interacting proteins were significantly reduced. Additionally, levels of aldo-keto reductase family 1 members A1 (P14550), B1 (P15121), B10 (O60218), and C3 (A0A0A0MSS8) were significantly increased in the UVB group. Overall, while UVB + CBD treatment resulted in an additional increase in these enzymes, there was a reduction in their levels in the CBD + UVB + CBD group. However, in the case of other antioxidant enzymes—carbonyl reductase (NADPH)1 (P16152), NAD(P)H quinone 1 dehydrogenase (P15559), and peroxiredoxin-1 (Q06830)–short-term CBD treatment (UVB + CBD) increased upon their UVB-elevated levels. Conversely, UVB-increased levels of these enzymes were reduced (or were less elevated) than in the CBD + UVB + CBD group. In addition, a large increase in the level of these antioxidant enzymes was observed in the CBD(24 h) group, and either a reduction, no change, or only a slight increase was observed in the CBD(48 h) group compared to the CTR group (Table S2).

Moreover, it has also been observed that UVB radiation increased the level of the S100 protein (V9HWH9), especially the S100-A6 protein (P06703), in comparison to the CTR cells. These UVB-increased levels were maintained in the UVB + CBD group. However, elevated levels of S100/-A6 protein were reduced in the CBD + UVB + CBD group. In addition, it was observed that a short application of CBD(24 h) induced the level of these proteins, while the levels of these proteins did not change significantly (S100-A6) or slightly changed (S100) in the CBD(48 h) group (Table S2).

**Figure 6:**
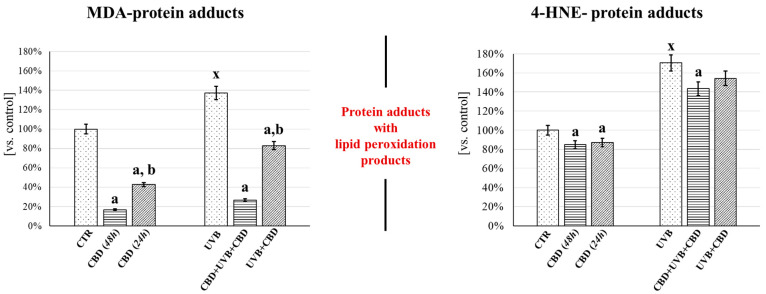
Effect of CBD and UVB radiation on the formation of adducts of proteins with lipid peroxidation products (MDA and 4-HNE) in keratinocyte membrane fractions. Keratinocytes were prepared as follows: [CTR]—cultured with standard medium; [CBD(48 h)]‚—cultured in medium containing 4 µM CBD for 48 h; [CBD(24 h)]—cultured in medium containing 4 µM CBD for 24 h; [UVB]—irradiated with UVB (312 nm) at 60 mJ/cm^2^ on standard medium, [CBD + UVB + CBD]—cultured with medium containing 4 μM CBD 24 h before and after UVB irradiation; [UVB + CBD]—cultured with medium containing 4 μM CBD 24 h after UVB irradiation. Mean values ± SD of five independent samples and statistically significant differences for *p* ≤ 0.05 are presented: a—differences vs. CTR or UVB; b—differences vs. CBD(48 h) or CBD + UVB + CBD; x—differences between UVB and CTR group.

Regardless of the changes in the level of proteins under the influence of both UVB radiation and CBD treatment, changes were found in the level of adducts formed between the analyzed proteins and lipid peroxidation products (4-HNE and MDA). It was shown that the use of CBD (both UVB + CBD and CBD + UVB + CBD) caused a reduction in UVB-elevated levels of 4-HNE-protein and MDA-protein adducts. Additionally, long-term application of CBD (CBD + UVB + CBD) led to a greater reduction in the level of protein adducts with MDA than with 4-HNE. At the same time, it was found that CBD (both CBD(24 h) and CBD(48 h)) reduced the level of MDA-protein adducts more effectively than the level of 4-HNE-protein adducts (Figure 6).

Detailed analysis of the MDA/4-HNE modified proteins showed interesting relationships (Tables S3 and S4). The acidic leucine-rich nuclear phosphoprotein belonging to the B family (Q92688) was found to form adducts with MDA only in the CBD + UVB + CBD cell group (Table S3). However, some ribosomal proteins were modified by 4-HNE including 40S ribosomal protein S7 (P62081) and 60S ribosomal protein L35a (P18077) in the UVB + CBD group and 40S ribosomal protein SA (A0A0C4DG17) in the CBD + UVB + CBD group. On the other hand, the antioxidant enzymes peroxiredoxins -1, -4, -5, and -6 (Q06830, A6NJJ0, P30044, P30041) were found in the form of adducts with 4-HNE in both groups irradiated with UVB and treated with CBD (UVB + CBD, CBD + UVB + CBD, and UVB). In the CBD(24 h) group, adducts of peroxiredoxin-4 (A6NJJ0) with 4-HNE were also found. In contrast, in the groups treated with both UVB and CBD, adducts of the beta proteasome subunit (A0A384NL22) with 4-HNE were found as well as adducts of several proteases (sentrin-specific protease 6 (Q9GZR1), transmembrane protease serine 4 (Q9NRS4), 11E (Q9UL52), and 13 (Q9BYE2) as well as mannan-binding lectin serine protease 1 (P48740)) with both MDA and with 4-HNE, simultaneously.

## 4. Discussion

The epidermis, the basic cells of which are keratinocytes, is an effective barrier that protects the organism against physical and chemical environmental stressors including UV radiation [22]. Every day, the skin is exposed to UVB radiation, which is a component of sunlight. This high-energy radiation is absorbed mainly by the epidermis, and its action promotes the development of a pro-inflammatory response and the formation of oxidative stress, promoting damage to DNA and other biologically important cell compounds, which may lead to the development of various skin diseases including cancer [23]. Cell membrane components (including phospholipids and proteins that are responsible for maintaining the stability of the composition and functionality of keratinocytes) are particularly exposed to UV radiation. The integrity of cell membranes ensures proper metabolic and functional activity of keratinocytes including intra- and intercellular signaling, which determines the transmission of important information in cells and to the deeper layers of the skin/organism [24]. Earlier in vivo and in vitro studies have shown that UVB radiation qualitatively and quantitatively modifies keratinocyte components including cellular proteins [4,25,26]. Through the increased generation of ROS, UVB radiation promotes ROS- and enzyme-dependent modifications of the metabolism of membrane phospholipids [15,27,28]. ROS-dependent modifications lead to significant increase in the generation of extremely reactive lipid peroxidation products (α,β non-saturated aldehydes and prostaglandin derivatives) and reduce the level of sialic acid, resulting in disturbance of cell membrane integrity [15,29]. Moreover, it has been indicated that UVB radiation leads to the induction of activity of ABC transmembrane transporters [15] as well as bilitranslocase [30]. However, another study showed that UVB radiation could significantly reduce the levels of Rac1 and atypical active forms of protein kinase C, which are involved in the continuity of tight junctions by controlling the permeability of cell membranes [31]. In addition, it is known that UVB radiation enhances protein carbonylation and keratinocyte proliferation [32] as well as increases the formation of adducts of proteins with lipid peroxidation products, mainly 4-HNE, which are involved in molecular transport and transduction [33].

Consequently, by modifying cell membrane components, UVB radiation induces depolarization of the mitochondrial membrane, which may mediate the induction of cell death [34]. Moreover, changes in redox status as well as inflammation resulting from changes in the expression of transcription factors (including the interacting pair Nrf2 and NF-κB) modify cellular metabolism [14]. This modulates the levels of cytoprotective proteins such as thioredoxin and glutaredoxin [35], and pro-inflammatory mediators such as TNF-α [Na, 2018]. Data from the literature [15,27,30,36] as well as the results obtained in this study show that UVB also affects biological membranes of keratinocytes regardless of the modification of membrane lipid components [37,38]. UVB radiation has been shown to modify important membrane proteins including antioxidant, apoptosis-related, proteasomal, and calcium-ion-binding proteins. Accordingly, the use of CBD has proved beneficial for the proper expression of these proteins in UVB-irradiated keratinocytes. In addition, since previous studies have shown that the course of action and effectiveness of CBD depend on the duration of its use, and that applying CBD before and after irradiation is more effective in reducing the negative effects of UVB radiation [15], this study compared both methods of CBD application.

The results of this study show that, depending on the duration of application, CBD modifies redox balance, either preventing a decrease or intensifying a decrease in the level of antioxidant proteins, which in turn modulates the level of oxidative stress and the level of lipid peroxidation products (MDA and 4-HNE) formed as a result of UVB-dependent stress. Consequently, this lowers the level of protein adducts with these lipid peroxidation products. The use of CBD has been shown to enhance the level of cytoprotective proteins from the aldo-keto reductase family 1 (A1, B1, B10, C3) [39], the transcription of which is regulated by a signaling pathway mediated by Nrf2, which provides living organisms with an effective defense against endogenous and environmental stressors, as demonstrated previously [40]. Moreover, cells treated with CBD only after UVB irradiation showed a higher increase of the above-mentioned enzymes than keratinocytes treated with CBD both before and after UVB irradiation. We also found such a tendency in another antioxidant protein, NAD(P)H (quinone) dehydrogenase 1, which exhibits both superoxide oxidoreductase and dismutase activity by participating in the regulation of redox homeostasis [41] and promotes the accumulation of p53 protein which, as a transcriptional activator, plays a role in apoptosis induction [42]. A previous study showed that overexpression of this enzyme can promote apoptosis in hepatocellular carcinoma cells and inhibit proliferation [43]. On the other hand, exposure of keratinocytes to CBD before and after UVB can cause the opposite effect, intensifying the pro-apoptotic effects, which may indicate the protection of keratinocytes (e.g., against pathological cell proliferation and carcinogenesis), as shown previously [44,45]. Taking into account the above, and the fact that long-term application of CBD reduces the severity of the antioxidant response, this may indicate a pro-apoptotic effect of this CBD application. Additionally, the increase in the levels of peroxiredoxin −1, −4, −5, and −6 adducts with 4-HNE in cells treated with CBD and UVB observed in this study may indicate dysfunction of these enzymes [46], which, according to the literature [47], may intensify pro-apoptotic signaling.

Keratinocytes treated with CBD after UVB irradiation are also characterized by an increase in the levels of other antioxidant enzymes such as carbonyl reductase 1 and peroxiredoxin-1, while the use of CBD before and after UVB irradiation decreases the levels of the above-mentioned enzymes. However, carbonyl reductase 1, a member of the NADPH-dependent short-chain dehydrogenase/reductase superfamily, is known to play a key role in cell survival under oxidative stress due to its antioxidant activity [48]. On the other hand, peroxiredoxin-1, in addition to its antioxidant effect, can inhibit ROS-induced apoptosis [49]. Moreover, this enzyme exhibits anti-inflammatory properties due to its interaction with the DNA repair protein APE1, which may affect the efficiency of DNA-NF-κB binding [50]. However, it has been shown that inhibition of APE1 activity also increases the level of Nrf2 target genes in pancreatic cancer cell lines [51]. The results of this study showed that the antioxidant and anti-inflammatory responses of keratinocytes to oxidative stress induced by UVB radiation can be induced by application of CBD only after UVB irradiation. A similar trend to lower the expression of antioxidant proteins (Nrf2 and superoxide dismutase) as well as proteins involved in the regulation of NF-κB signaling such as arginine succinate synthase was observed in rat skin keratinocytes exposed to UVA/B radiation and CBD treatment for 28 days [25]. It can be suggested that the differentiation of the metabolic response of keratinocytes to different periods of CBD application may confirm its dependence simultaneously on two transcription factors (Nrf2 and NF-κB) that interact with each other [14,52]. Consequently, CBD can modify both antioxidant and inflammatory effects as well as influence other metabolic reactions, as described below.

Oxidative stress induced by UVB radiation promotes the formation of protein adducts with MDA and 4-HNE, which may alter protein folding, function, and even modulation of various intra- and intercellular signaling pathways [33,53]. Since long-term use of CBD does not reduce the level of protein-4-HNE adducts as effectively as in the case of protein-MDA adducts, the changes observed after CBD exposure can be interpreted as a positive effect of CBD on the survival of keratinocytes, as the presence of MDA epitopes in apoptotic cells has been demonstrated previously (adducts resulting from covalent modification of primary amino groups in proteins and lipids) [54]. The literature data clearly indicate that, due to the ability of 4-HNE to form protein adducts and promote oxidative stress (especially in the mitochondria), 4-HNE can indeed be considered a special inducer of apoptosis [55].

Moreover, another target for modification by 4-HNE is the ubiquitin-related proteasomal system, wherein the intensive modification combined with the cross-linking of protein molecules may lead to the formation of protein aggregates resistant to degradation and thus inhibit the proteasomal system [56]. Aggregation may also be promoted by Ca^2+^ ion accumulation [57] and the resulting aggregates may disrupt cellular electrolytic homeostasis and even induce an apoptotic response that may be accompanied by the formation of pores in the membranes [57]. The results of this work showed a significant increase in the level of 4-HNE adducts with the beta subunit of the proteasome in keratinocyte membranes subjected to UVB and long-term CBD application. There are no literature data on the influence of such modifications on the biological activity of this protein, but taking into account similar situations in other conditions, a reduction in proteolytic efficiency may be suggested. However, long-term application of CBD combined with UVB irradiation also favors the formation of adducts of MDA with the acidic, leucine-rich nuclear phosphoprotein from the B 32 family, which has been identified as an anti-apoptotic protein in leukemic cells [58]. It can therefore be suggested that CBD, by modifying the level of protein adducts with lipid peroxidation products, may alter the cellular response to pro-oxidative factors (UVB) by regulating proteostasis and intracellular signaling including apoptosis.

The above changes are also accompanied by an increase in pro-apoptotic ribosomal proteins in the membranes of CBD-treated keratinocytes before and after UVB irradiation. It can be concluded that CBD increases the UVB-induced levels of 40S ribosomal proteins (S15a, S3a), all of which may promote cell differentiation, neoplastic transformation, and anti-apoptotic activity [59]. At the same time, however, there is also a significant increase in the level of other ribosomal proteins such as the 40S ribosomal proteins S3 and S19 (involved in the direct/indirect regulation of the tumor suppressor protein p53) as well as the 60S ribosomal protein and the proteins L13a and L7a, which can induce apoptosis through participation in the cell cycle and translation by accumulation of the p53 protein [60,61]. Since UVB radiation is one of primary reasons for malignant transformation in skin cells, it can be suggested that CBD, through its probable pro-apoptotic effect, can protect cells against metabolic changes caused by UVB, which are unfavorable for the cell, as they may, for example, lead to neoplastic transformation.

In contrast, our previous study, in vivo, showed, that CBD was able to reverse UV-induced pro-apoptotic pathways by modifying anti-apoptotic and pro-apoptotic factors such as the apoptotic regulator Bcl-2 in the cytosol of keratinocytes from UVA/UVB-irradiated skin of nude rats [25]. It is known, however, that not only does the quantitative level of Bcl-2 determine the metabolic importance of CBD, but post-translational modifications also affect the stability, localization, and function of this protein, and this may ultimately enhance or inhibit apoptosis [62]. It is also known that the level of NF-κB and the signaling pathways it promotes are important for the induction of apoptosis. It has been shown, inter alia, that there is an interaction between the S3 protein and the IκBα inhibitor NF-κB in HEK293 cells [63,64]. Moreover, it was found that the S3 protein can bind to the p65 subunit of NF-κB, which promotes accumulation in the nucleus and increases the binding affinity of the NF-κB complex. Thus, CBD can induce the expression of pro-inflammatory proteins, possibly by activating the NF-κB transcription factor and/or modulating the interaction of Nrf2 and NF-κB, as previously demonstrated [25]. Such a situation may trigger the activation of elimination processes of proteins with a pathologically altered structure, which may interfere with physiological processes in keratinocytes. Moreover, the pro-apoptotic effect of CBD, through its demonstrated tendency to lower peroxiredoxin-1 levels and possibly also through its modification by 4-HNE, may also support the long-term proliferation protection effect of keratinocytes. This situation is enhanced by the increased level of pro-apoptotic factors as above-mentioned such as the ribosomal protein L7a [61].

In the regulation of cell survival including growth, differentiation, and metabolism, Ca^2+^ ions are also involved [65], which simultaneously play a key role in apoptosis, but also in the anti-inflammatory response and in driving phagocytes to apoptosis [66,67]. The present results indicate that UVB radiation increased the level of the protein S100-A6, which is involved in the regulation of calcium-binding proteins, cell proliferation, and apoptosis [68]. Thus, there is big interest in the S100 family members because of their potential crucial role in tumor development and progression [69]. The literature indicates that the transcription factors Nrf2 and NF-κB activate the S100-A6 gene promotor [68]; therefore, the increase in the level of S100-A6 caused by CBD may be a result of the increase in the expression of the above transcription factors by this phytocannabinoid. Although an anti-apoptotic effect of S100-A6 was found in the literature, some studies also showed that S100-A6 can favor apoptosis in HEp-3B cells [70] and HEp-2 cells [71]. Moreover, it has been indicated that S100-A6 can trigger neuronal apoptosis by causing ROS-dependent activation of JNK and of caspases −3 and −7 in SH-SY5Y neuroblastoma cells [72]. Therefore, the suggested tendency to modify cell metabolism toward apoptosis may also be related to the effect of CBD on the level/activity of S100-A6.

## 5. Conclusions

This study showed that CBD affects the membrane proteins of keratinocytes exposed to UVB radiation in various ways, and may suggest that the observed metabolic response is time-dependent in relation to CBD application. CBD used only after UVB irradiation enhances the antioxidant response initiated by UVB radiation. Despite the increase in the proteasomal degradation efficiency upon the modified proteins, their level is not completely reduced, and the accumulation of adducts of protein-lipid peroxidation products is constantly observed. In contrast, when CBD is used before and after UVB irradiation of cells, the accumulation of modified proteins may occur due to the reduced efficiency of proteasomes (possibly with increased protein aggregation), which disrupts signaling pathways in keratinocytes, and thus leads to activation of proteins involved in the process of apoptosis. Thus, with both short-term and long-term use of CBD, this phytocannabinoid triggers cellular protective mechanisms related to either the degradation of modified proteins or the apoptosis of pathologically altered cells.

## Data Availability

The data presented in this study are available in supplementary material.

## Supplementary Materials

The following are available online at https://www.mdpi.com/2076-3921/10/3/402/s1.

## References

[1] Matsumura Y., Ananthaswamy H.N. (2004). Toxic effects of ultraviolet radiation on the skin. Toxicol. Appl. Pharmacol..

[2] Orazio D.J., Jarrett S., Ortiz A.A., Scott T. (2013). UV Radiation and the Skin. Int. J. Mol. Sci..

[3] Schuch A.P., Moreno N.C., Schuch N.J., Menck C.F.M., Garcia C.C.M. (2017). Sunlight damage to cellular DNA: Focus on oxidatively generated lesions. Free Radic. Biol. Med..

[4] Zhu X., Li N., Wang Y., Ding L., Chen H., Yu Y., Shi X. (2016). Protective effects of quercetin on UVB irradiation-induced cytotoxicity through ROS clearance in keratinocyte cells. Oncol. Rep..

[5] Ortiz A.A., Yan B., Orazio O.J.A. (2014). Ultraviolet Radiation, Aging and the Skin: Prevention of Damage by Topical cAMP Manipulation. Molecules.

[6] Rastogi R.P., Kumar A., Tyagi M.B., Sinha R.P. (2010). Molecular mechanisms of ultraviolet radiation-induced DNA damage and repair. J. Nucleic Acids.

[7] Qin J.Z., Chaturvedi V., Denning M.F., Bacon P., Panella J., Choubey D., Nickoloff B.J. (2002). Regulation of apoptosis by p53 in UV-irradiated human epidermis, psoriatic plaques and senescent keratinocytes. Oncogene.

[8] Coelho S.G., Choi W., Brenner M., Miyamura Y., Yamaguchi Y., Wolber R., Smuda C., Batzer J., Kolbe L., Ito S. (2009). Short- and Long-Term Effects of UV Radiation on the Pigmentation of Human Skin. J. Investig. Dermatol. Symp. Proc..

[9] Oliveira M.M., Ratti B.A., Daré R.G., Silva S.O., Truiti M.D.C.T., Nakamura U.T., Velty A.R., Nakamura C.V. (2019). Dihydrocaffeic Acid Prevents UVB-Induced Oxidative Stress Leading to the Inhibition of Apoptosis and MMP-1 Expression via p38 Signaling Pathway. Oxidative Med. Cell. Longev..

[10] Kremslehner C., Miller A., Nica R., Nagelreiter I.M., Narzt M.S., Golabi B., Vorstandlechner V., Mildner M., Lachner J., Tschachler E. (2020). Imaging of metabolic activity adaptations to UV stress, drugs and differentiation at cellular resolution in skin and skin equivalents–Implications for oxidative UV damage. Redox Biol..

[11] Martínez V., De Hond A.I., Borrelli F., Capasso R., Del Castillo M.D., Abalo R. (2020). Cannabidiol and Other Non-Psychoactive Cannabinoids for Prevention and Treatment of Gastrointestinal Disorders: Useful Nutraceuticals?. Int. J. Mol. Sci..

[12] Atalay S., Karpowicz J.I., Skrzydlewska E. (2019). Antioxidative and Anti-Inflammatory Properties of Cannabidiol. Antioxidants.

[13] Di Giacomo V., Recinella L., Chiavaroli A., Orlando G., Cataldi A., Rapino M., Di Valerio V., Politi M., Antolini M.D., Acquaviva A. (2021). Metabolomic profile and antioxidant/anti-inflammatory effects of industrial hemp water extract in fi-broblasts, keratinocytes and isolated mouse skin specimens. Antioxidants.

[14] Jastrząb A., Gęgotek A., Skrzydlewska E. (2019). Cannabidiol Regulates the Expression of Keratinocyte Proteins Involved in the Inflammation Process through Transcriptional Regulation. Cells.

[15] Atalay S., Dobrzyńska I., Gęgotek A., Skrzydlewska E. (2020). Cannabidiol protects keratinocyte cell membranes following exposure to UVB and hydrogen peroxide. Redox Biol..

[16] Bradford M. (1976). A Rapid and Sensitive Method for the Quantitation of Microgram Quantities of Protein Utilizing the Principle of Protein-Dye Binding. Anal. Biochem..

[17] Gęgotek A., Domingues P., Wroński A., Wójcik P., Skrzydlewska E. (2018). Proteomic plasma profile of psoriatic patients. J. Pharm. Biomed. Anal..

[18] Tyanova S., Temu T., Sinitcyn P., Carlson A., Hein M.Y., Geiger T., Mann M., Cox J. (2016). The Perseus computational platform for comprehensive analysis of (prote)omics data. Nat. Methods.

[19] Chong J., Soufan O., Li C., Caraus I., Li S., Bourque G., Wishart D.S., Xia J. (2018). MetaboAnalyst 4.0: Towards more trans-parent and integrative metabolomics analysis. Nucleic Acids Res..

[20] Thomas P.D., Campbell M.J., Kejariwal A., Mi H., Karlak B., Daverman R., Diemer K., Muruganujan A., Narechania A. (2003). PANTHER: A Library of Protein Families and Subfamilies Indexed by Function. Genome Res..

[21] Szklarczyk D., Gable A.L., Lyon D., Junge A., Wyder S., Cepas H.J., Simonovic M., Doncheva N.T., Morris J.H., Bork P. (2019). STRING v11: Protein–protein association networks with increased coverage, supporting functional discovery in genome-wide experimental datasets. Nucleic Acids Res..

[22] Parrado C., Saenz M.S., Davo P.A., Gilaberte Y., Gonzalez S., Juarranz Á. (2019). Environmental Stressors on Skin Aging. Mechanistic Insights. Front. Pharmacol..

[23] Umar S.A., Tasduq S.A. (2020). Integrating DNA damage response and autophagy signalling axis in ultraviolet-B induced skin photo-damage: A positive association in protecting cells against genotoxic stress. RSC Adv..

[24] Watson H. (2015). Biological membranes. Essays Biochem..

[25] Atalay S., Gęgotek A., Wroński A., Domigues P., Skrzydlewska E. (2021). Therapeutic application of cannabidiol on UVA and UVB irradiated rat skin. A proteomic study. J. Pharm. Biomed. Anal..

[26] Khalil C., Shebaby W. (2017). UVB damage onset and progression 24 h post exposure in human-derived skin cells. Toxicol. Rep..

[27] Karpowicz J.I., Biernacki M., Wroński A., Gęgotek A., Skrzydlewska E. (2020). Cannabidiol Effects on Phospholipid Metabolism in Keratinocytes from Patients with Psoriasis Vulgaris. Biomolecules.

[28] Łuczaj W., Domingues M.D.R., Domingues P., Skrzydlewska E. (2020). Changes in Lipid Profile of Keratinocytes from Rat Skin Exposed to Chronic UVA or UVB Radiation and Topical Application of Cannabidiol. Antioxidants.

[29] Dobrzyńska I., Petelska S.B., Wroński A., Karpowicz J.I., Skrzydlewska E. (2020). Changes in the physico-chemical properties of blood and skin cell membranes as a result of psoriasis vulgaris and psoriatic arthritis development. Int. J. Mol. Sci..

[30] Gęgotek A., Ambrożewicz E., Jastrząb A., Karpowicz J.I., Skrzydlewska E. (2019). Rutin and ascorbic acid cooperation in antioxidant and antiapoptotic effect on human skin keratinocytes and fibroblasts exposed to UVA and UVB radiation. Arch. Dermatol. Res..

[31] Yuki T., Hachiya A., Kusaka A., Sriwiriyanont P., Visscher M.O., Morita K., Muto M., Miyachi Y., Sugiyama Y., Inoue S. (2011). Characterization of Tight Junctions and Their Disruption by UVB in Human Epidermis and Cultured Keratinocytes. J. Investig. Dermatol..

[32] Wang P.W., Hung Y.C., Lin T.Y., Fang J.Y., Yang P.M., Chen M.H., Pan T.L. (2019). Comparison of the Biological Impact of UVA and UVB upon the Skin with Functional Proteomics and Immunohistochemistry. Antioxidants.

[33] Gęgotek A., Atalay S., Domingues P., Skrzydlewska E. (2019). The Differences in the Proteome Profile of Cannabidiol-Treated Skin Fibroblasts following UVA or UVB Irradiation in 2D and 3D Cell Cultures. Cells.

[34] Jugé R., Breugnot J., Da Silva C., Bordes S., Closs B., Aouacheria A. (2016). Quantification and Characterization of UVB-Induced Mitochondrial Fragmentation in Normal Primary Human Keratinocytes. Sci. Rep..

[35] Gęgotek A., Karpowicz J.I., Skrzydlewska E. (2020). Cytoprotective Effect of Ascorbic Acid and Rutin against Oxidative Changes in the Proteome of Skin Fibroblasts Cultured in a Three-Dimensional System. Nutrients.

[36] Perluigi M., Di Domenico F., Blarzino C., Foppoli C., Cini C., Giorgi A., Grillo C., De Marco F., Butterfield D.A., Schininà M.E. (2010). Effects of UVB-induced oxidative stress on protein expression and specific protein oxidation in normal human epithelial keratinocytes: A proteomic approach. Proteome Sci..

[37] Pichler H., Augustin E.A. (2018). Modification of membrane lipid compositions in single-celled organisms—From basics to applications. Methods.

[38] Casares D., Escribá P.V., Rosselló C.A. (2019). Membrane lipid composition: Effect on membrane and organelle structure, function and compartmentalization and therapeutic avenues. Int. J. Mol. Sci..

[39] Penning T.M. (2017). Aldo-Keto Reductase Regulation by the Nrf2 System: Implications for Stress Response, Chemotherapy Drug Resistance, and Carcinogenesis. Chem. Res. Toxicol..

[40] Huang Y., Li W., Su Z.Y., Kong A.N.T. (2015). The complexity of the Nrf2 pathway: Beyond the antioxidant response. J. Nutr. Biochem..

[41] Ross D., Siegel D. (2017). Functions of NQO1 in Cellular Protection and CoQ10 Metabolism and its Potential Role as a Redox Sensitive Molecular Switch. Front. Physiol..

[42] Liu K., Jin B., Wu C., Yang J., Zhan X., Wang L., Shen X., Chen J., Chen H., Mao Z. (2015). NQO1 Stabilizes p53 in Response to Oncogene-Induced Senescence. Int. J. Biol. Sci..

[43] Zhang X., Han K., Yuan D.H., Meng C.Y. (2017). Overexpression of NAD(P)H: Quinone Oxidoreductase 1 Inhibits Hepatocel-lular Carcinoma Cell Proliferation and Induced Apoptosis by Activating AMPK/PGC-1α Pathway. DNA Cell Biol..

[44] Shrivastava A., Kuzontkoski P.M., Groopman J.E., Prasad A. (2011). Cannabidiol Induces Programmed Cell Death in Breast Cancer Cells by Coordinating the Cross-talk between Apoptosis and Autophagy. Mol. Cancer Ther..

[45] Lukhele S.T., Motadi L.R. (2016). Cannabidiol rather than Cannabis sativa extracts inhibit cell growth and induce apoptosis in cervical cancer cells. BMC Complement. Altern. Med..

[46] Csala M., Kardon T., Legeza B., Lizák B., Mandl J., Margittai É., Puskás F., Száraz P., Szelényi P., Bánhegyi G. (2015). On the role of 4-hydroxynonenal in health and disease. Biochim. Biophys. Acta (BBA) Mol. Basis Dis..

[47] Feng H., Li Z., Du J., Sun J., Feng W., Li D., Liu S., Wang W., Liu H., Amizuka N. (2018). Dual function of peroxiredoxin I in lipopolysaccharide-induced osteoblast apoptosis via reactive oxygen species and the apoptosis signal-regulating kinase 1 signaling pathway. Cell Death Discov..

[48] Kim Y.N., Jung H.Y., Eum W.S., Kim D.W., Shin M.J., Ahn E.H., Kim S.J., Lee C.H., Yong J.I., Ryu E.J. (2014). Neuro-protective effects of PEP-1-carbonyl reductase 1 against oxidative-stress-induced ischemic neuronal cell damage. Free Radic. Biol. Med..

[49] Han B., Shin H.J., Bak I.S., Bak Y., Jeong Y.L., Kwon T., Park Y.H., Sun H.N., Kim C.H., Yu D.Y. (2016). Peroxiredoxin I is important for cancer-cell survival in Ras-induced hepatic tumorigenesis. Oncotarget.

[50] Nassour H., Wang Z., Saad A., Papaluca A., Brosseau N., Affar E.B., Jamali A.M.A., Ramotar D. (2016). Peroxiredoxin 1 interacts with and blocks the redox factor APE1 from activating interleukin-8 expression. Sci. Rep..

[51] Tebay L.E., Robertson H., Durant S.T., Vitale S.R., Penning T.M., Kostova D.A.T., Hayes J.D. (2015). Mechanisms of activation of the transcription factor Nrf2 by redox stressors, nutrient cues, and energy status and the pathways through which it attenuates degenerative disease. Free Radic. Biol. Med..

[52] Wardyn J.D., Ponsford A.H., Sanderson C.M. (2015). Dissecting molecular cross-talk between Nrf2 and NF-κB response pathways. Biochem. Soc. Trans..

[53] Maier C.S., Chavez J., Wang J., Wu J. (2010). Protein Adducts of Aldehydic Lipid Peroxidation Products: Identification and Characterization of Protein Adducts Using an Aldehyde/Keto-Reactive Probe in Combination with Mass Spectrometry. Methods in Enzymology.

[54] Hyvärinen S., Uchida K., Varjosalo M., Jokela R., Jokiranta T.S. (2014). Recognition of Malondialdehyde-modified Proteins by the C Terminus of Complement Factor H Is Mediated via the Polyanion Binding Site and Impaired by Mutations Found in Atypical Hemolytic Uremic Syndrome. J. Biol. Chem..

[55] Dalleau S., Baradat M., Guéraud F., Huc L. (2013). Cell death and diseases related to oxidative stress:4-hydroxynonenal (HNE) in the balance. Cell Death Differ..

[56] Castro J.P., Jung T., Grune T., Siems W. (2017). 4-Hydroxynonenal (HNE) modified proteins in metabolic diseases. Free Radic. Biol. Med..

[57] Dash R., Ali C., Jahan I., Munni Y.A., Mitra S., Hannan A., Timalsina B., Oktaviani D.F., Choi H.J., Moon I.S. (2021). Emerging potential of cannabidiol in reversing proteinopathies. Ageing Res. Rev..

[58] Yu Y., Shen S.M., Zhang F.F., Wu Z.X., Han B., Wang L.S. (2012). Acidic leucine-rich nuclear phosphoprotein 32 family member B (ANP32B) contributes to retinoic acid-induced differentiation of leukemic cells. Biochem. Biophys. Res. Commun..

[59] Zhou X., Liao W.J., Liao J.M., Liao P., Lu H. (2015). Ribosomal proteins: Functions beyond the ribosome. J. Mol. Cell Biol..

[60] Chen F.W., Ioannou Y.A. (1999). Ribosomal Proteins in Cell Proliferation and Apoptosis. Int. Rev. Immunol..

[61] Xu X., Xiong X., Sun Y. (2016). The role of ribosomal proteins in the regulation of cell proliferation, tumorigenesis, and genomic integrity. Sci. China Life Sci..

[62] Kale J., Osterlund E.J., Andrews D.W. (2018). BCL-2 family proteins: Changing partners in the dance towards death. Cell Death Differ..

[63] Wan F., Anderson D.E., Barnitz R.A., Snow A., Bidere N., Zheng L., Hegde V., Lam L.T., Staudt L.M., Levens D. (2007). Ribosomal Protein S3: A KH Domain Subunit in NF-κB Complexes that Mediates Selective Gene Regulation. Cell.

[64] Stanborough T., Niederhauser J., Koch B., Bergler H., Pertschy B. (2014). Ribosomal protein S3 interacts with the NF-κB inhibitor IκBα. FEBS Lett..

[65] Varghese E., Samuel S.M., Sadiq Z., Kubatka P., Liskova A., Benacka J., Pazinka P., Kruzliak P., Büsselberg D. (2019). Anti-cancer agents in proliferation and cell death: The calcium connection. Int. J. Mol. Sci..

[66] Rizzuto R., Pinton P., Ferrari D., Chami M., Gy O., Szabadkai R., Magalhães P.J. (2003). Calcium and apoptosis: Facts and hypotheses. Oncogene.

[67] Gronski M.A., Kinchen J.M., Juncadella I.J., Franc N.C., Ravichandran K.S. (2009). An essential role for calcium flux in phag-ocytes for apoptotic cell engulfment and the anti-inflammatory response. Cell Death Differ..

[68] Donato R., Sorci G., Giambanco I. (2017). S100A6 protein: Functional roles. Cell. Mol. Life Sci..

[69] Allgöwer C., Kretz A.L., Von Karstedt S., Wittau M., Bruns H.D., Lemke J. (2020). Friend or Foe: S100 Proteins in Cancer. Cancers.

[70] Joung H.J., Sun Y.Y., Joo H.K., Paik S.G., Sung R.M., Lim J.S., In S.C., Choi I., Jae W.K. (2008). S100A6 (calcyclin) enhances the sensitivity to apoptosis via the upregulation of caspase-3 activity in Hep3B cells. J. Cell. Biochem..

[71] Słomnicki Ł.P., Nawrot B., Leśniak W. (2009). S100A6 binds p53 and affects its activity. Int. J. Biochem. Cell Biol..

[72] Leclerc E., Fritz G., Weibel M., Heizmann C.W., Galichet A. (2007). S100B and S100A6 Differentially Modulate Cell Survival by Interacting with Distinct RAGE (Receptor for Advanced Glycation End Products) Immunoglobulin Domains. J. Biol. Chem..

