# Supplementary material for: Protective Effects of Cannabidiol on the Membrane Proteome of UVB-Irradiated Keratinocytes
Source: Antioxidants (Basel). 2021 Mar 8;10(3):402. doi: 10.3390/antiox10030402 (PMC8001542; doi:10.3390/antiox10030402)
Supplement: Supplementary file 1 [file antioxidants-10-00402-s001.pdf]

Table S1. ID, name, number of assigned as well as unique peptides, sequence coverage [%] and average intensity list of proteins identified in each experimental group: control group cultured with standard medium [**CTR**]; cell group cultured with standard medium containing 4 μM CBD for 48 h [**CBD (48h)**]; cell group cultured with standard medium containing 4 μM CBD for 24 h CBD [**CBD (24h)**]; cell group irradiated by UVB (312 nm) at 60 mJ/cm^2 [**UVB**]; cell group cultured with standard medium containing 4 μM CBD for 24 h before and after UVB irradiation [**CBD+UVB+CBD**]; cell group cultured with standard medium containing 4 μM CBD for 24 h only after UVB irradiation [**UVB+CBD**]. Empty cells indicate that "this protein was not identified in related sample".

ID	Protein name	Number of assigned peptides	Number of unique peptides	Sequence coverage [%]	CTR	CBD (48h)	CBD (24h)	UVB	CBD+UVB+CBD	UVB+CBD
**A0A023T6R1**	Mago nashi protein	2	2	12.16216216						6.00E+06
**A0A024QZ77**	EF-hand domain family, member D2	4	4	17.91666667			3.70E+06	4.70E+06	8.30E+06	7.53E+06
**A0A024QZK8**	Heterogeneous nuclear ribonucleoprotein H3 (2H9)	4	4	13.87283237		8.20E+05		4.30E+06		8.35E+06
**A0A024QZN9**	Outer mitochondrial membrane protein porin 2	7	6	27.5862069		4.70E+05	5.40E+06	5.35E+06	1.40E+07	1.37E+07
**A0A024QZT9**	NAD(P)H dehydrogenase, quinone 2	3	3	13.85281385			2.70E+06			4.80E+06
**A0A024R172**	15-oxoprostaglandin 13-reductase	10	10	42.24924012	3.49E+06	2.60E+06	1.43E+07	1.27E+07	2.82E+07	3.13E+07
**A0A024R1K7**	Tyrosine 3-monooxygenase/tryptophan 5-monooxygenase activation protein, eta polypeptide	11	8	37.39837398	9.20E+05	4.90E+06	6.50E+06	7.70E+05		3.30E+07
**A0A024R1N1**	Myosin, heavy polypeptide 9, non-muscle	47	47	30.56122449	7.00E+06	1.20E+06	1.10E+07	1.18E+07	9.93E+06	8.33E+06
**A0A024R1V4**	60S ribosomal protein L27	5	5	38.97058824	1.30E+06		1.07E+07	8.63E+06	3.20E+07	1.31E+07
**A0A024R1X8**	Junction plakoglobin	2	2	5.100671141			3.50E+06	3.50E+06		
**A0A024R231**	Guanine deaminase	22	22	68.50220264	9.77E+06	3.87E+06	2.52E+07	2.07E+07	2.15E+07	9.67E+06
**A0A024R2A7**	Lectin, mannose-binding, 1	2	2	3.725490196	2.50E+06					2.30E+05
**A0A024R2B6**	Serpin peptidase inhibitor, clade B (Ovalbumin), member 5	11	6	34.4	5.40E+06		2.40E+06		6.50E+06	7.80E+06
**A0A024R2Q4**	Ribosomal protein L15	4	4	17.15686275	9.40E+05		2.90E+06	2.80E+06	4.80E+05	7.55E+06

**A0A024R321**	Filamin B, beta (Actin binding protein 278)	67	61	36.30816171	4.31E+06	3.60E+06	1.85E+07	3.00E+07	1.60E+07	1.06E+07
**A0A024R3W7**	Elongation factor 1-beta	2	2	9.777777778			2.60E+05			
**A0A024R3Y6**	Isocitrate dehydrogenase [NADP]	26	26	67.87439614	1.42E+07	3.70E+06	2.62E+07	2.91E+07	3.19E+07	1.83E+07
**A0A024R454**	Carbamoyl-phosphate synthetase 1, mitochondrial	58	57	53.8	3.05E+07	3.83E+06	3.53E+07	3.13E+07	3.02E+07	2.46E+07
**A0A024R4K3**	Malate dehydrogenase	15	15	57.69230769	1.70E+07	3.30E+06	1.56E+07	1.75E+07	2.42E+07	4.50E+07
**A0A024R4U3**	Tubulin tyrosine ligase-like family, member 12	3	3	5.590062112	2.30E+06				3.30E+06	
**A0A024R5Z7**	Annexin	19	19	63.71681416	4.03E+06	4.67E+06	1.01E+07	1.25E+07	1.63E+07	3.07E+07
**A0A024R6D4**	Enhancer of rudimentary homolog	2	2	16.34615385	1.60E+06					4.20E+06
**A0A024R6Q1**	Eukaryotic translation initiation factor 5	5	5	15.0812065	1.40E+06	2.70E+06		1.40E+07	1.30E+07	6.20E+06
**A0A024R6S1**	Hsp40 homolog, subfamily A, member 2	2	2	9.708737864				2.10E+06	2.50E+06	6.30E+06
**A0A024R6W0**	Aspartate aminotransferase	15	15	41.1627907	1.20E+07	3.80E+06	1.39E+07	1.81E+07	1.71E+07	1.46E+07
**A0A024R6W2**	Cleavage and polyadenylation specificity factor subunit 5	2	2	19.38325991	2.40E+06	3.65E+06	5.00E+06	4.90E+06	1.30E+07	1.30E+07
**A0A024R6Y2**	Nuclear transport factor 2	2	2	18.8976378			1.30E+07	1.26E+07	1.70E+07	1.87E+07
**A0A024R718**	Nicotinamide phosphoribosyltransferase	31	31	66.598778	1.23E+07	1.03E+07	8.87E+07	7.78E+07	6.60E+07	5.50E+07
**A0A024R7M0**	Transmembrane emp24 protein transport domain containing 9	2	2	8.085106383						
**A0A024R814**	Ribosomal protein L7	14	14	49.80694981	2.00E+06	1.60E+06	5.70E+06	5.13E+06	1.50E+07	1.64E+07
**A0A024R8S5**	Protein disulfide-isomerase	37	37	75	1.60E+07	4.05E+06	3.03E+07	3.14E+07	2.77E+07	1.37E+07
**A0A024RAD5**	Dolichyl-diphosphooligosaccharide-protein glycosyltransferase 48 kDa subunit	3	3	6.798245614	3.90E+06					
**A0A024RAF2**	Diazepam binding inhibitor (GABA receptor modulator, acyl-Coenzyme A binding protein)	2	2	25.96153846						5.60E+06
**A0A024RAI1**	ARP3 actin-related protein 3 homolog	6	6	26.07655502	1.90E+06	1.10E+06	1.13E+07	1.60E+07	6.73E+06	6.69E+06

**A0A024RAM2**	Glutaredoxin (Thioltransferase)	2	2	37.73584906	2.90E+06	4.00E+06	8.30E+06	9.90E+06		
**A0A024RAZ7**	Helix-destabilizing protein	16	12	41.12903226	2.10E+06	5.00E+06	1.35E+07	2.20E+07	2.35E+07	2.40E+07
**A0A024RB75**	Citrate synthase	12	11	37.98283262	4.50E+06	1.95E+06	6.00E+06	7.90E+06	1.45E+07	8.60E+06
**A0A024RB87**	RAP1B, member of RAS oncogene family	3	3	13.58695652				6.30E+06		8.70E+06
**A0A024RBB7**	Nucleosome assembly protein 1-like 1	3	3	11.76470588				1.10E+07	1.80E+06	
**A0A024RBH2**	Cytoskeleton-associated protein 4	6	6	15.28239203			3.20E+06	3.20E+06	2.60E+06	
**A0A024RCN6**	Valyl-tRNA synthetase	8	8	8.860759494	3.35E+06				5.60E+06	2.05E+06
**A0A024RDL1**	Chaperonin containing TCP1, subunit 6A (Zeta 1)	10	10	24.10546139		2.17E+06	1.10E+07	6.10E+06	2.65E+06	9.37E+06
**A0A024RDR0**	High-mobility group box 1	8	8	33.95348837			1.50E+07	8.00E+06	1.10E+07	8.77E+06
**A0A024RDS1**	Heat shock 110 kDa protein	6	4	9.906759907		3.70E+06			1.30E+07	5.50E+06
**A0A087WTP3**	Far upstream element-binding protein 2	3	3	4.219409283		7.90E+05	1.00E+07	5.55E+06	7.00E+06	4.30E+06
**A0A087WUZ3**	Spectrin beta chain	32	32	17.58241758	2.80E+06	5.40E+05	6.10E+06	6.75E+06	4.52E+06	2.93E+06
**A0A087WVQ6**	Clathrin heavy chain	43	37	34.24657534	1.10E+07	2.11E+06	1.50E+07	1.13E+07	1.23E+07	8.83E+06
**A0A087WW66**	6S proteasome non-ATPase regulatory subunit 1	7	7	8.70933893	5.50E+06				8.50E+06	3.70E+06
**A0A087X0X3**	Heterogeneous nuclear ribonucleoprotein M	6	6	8.630136986			2.40E+06		1.20E+07	1.40E+07
**A0A087X1Z3**	Proteasome activator complex subunit 2	7	7	39.76377953	5.70E+05		2.60E+06	3.07E+06	4.10E+06	1.10E+07
**A0A087X2I1**	26S proteasome regulatory subunit 10B	3	3	10.91811414			5.00E+06	2.78E+06	4.55E+06	3.70E+06
**A0A0A0MRL7**	Caspase-7	2	2	10.56701031						7.10E+06
**A0A0A0MSE2**	Hydroxyacyl-coenzyme A dehydrogenase, mitochondrial	3	3	13.07692308	4.60E+06			1.40E+06	6.90E+06	7.20E+06
**A0A0A0MSS8**	Aldo-keto reductase family 1 member C3	21	8	63.46749226	4.10E+06	3.20E+06	4.80E+05	1.19E+07	2.28E+07	3.00E+07
**A0A0B4J269**	G protein receptor F1_2 domain-containing protein	14	3	19.32245922		5.50E+05		5.89E+06	2.87E+06	3.80E+06
**A0A0B4J2C3**	Translationally-controlled tumor protein	4	4	39.59390863			4.30E+06	7.10E+06	1.50E+07	7.13E+06
**A0A0C4DG17**	40S ribosomal protein SA	14	14	56	5.00E+06	5.35E+06	2.26E+07	2.39E+07	2.24E+07	1.75E+07

**A0A0C4DGQ5**	Calcium-activated neutral proteinase small subunit	3	3	18.01242236	9.40E+05			8.65E+06		
**A0A0D9SF53**	RNA helicase	11	3	20.05457026	3.30E+06	1.40E+06	1.90E+07	1.01E+07	4.60E+06	2.80E+06
**A0A0K0K1K4**	Proteasome subunit alpha type	12	12	47.17741935	5.90E+06	6.45E+06	8.68E+06	9.19E+06	1.35E+07	1.83E+07
**A0A0K0K1K7**	6-phosphogluconolactonase	3	3	17.82945736				8.80E+06		1.10E+07
**A0A0M4FEM1**	Alpha-1,4 glucan phosphorylase	9	3	13.89548694						5.55E+06
**A0A0S2Z3G9**	Actinin alpha 4 isoform 1	35	21	50.93304061	5.95E+06	1.60E+06	1.23E+07	1.47E+07	7.43E+06	3.63E+06
**A0A0S2Z3L0**	Electron transfer flavoprotein subunit alpha, mitochondrial	5	5	24.92492492	3.00E+06			1.35E+07		7.03E+06
**A0A0S2Z3Y1**	Galectin-3-binding protein	5	5	10.42735043	7.40E+06		4.35E+06	5.20E+06	1.06E+07	
**A0A0S2Z404**	Regulator of chromosome condensation 1 isoform 2	2	2	5.530973451				4.50E+06		
**A0A0S2Z471**	Creatine kinase	3	3	10.12345679			5.30E+06	8.80E+06	3.00E+06	1.60E+06
**A0A0S2Z489**	26S proteasome subunit, non-ATPase, 12	6	6	17.98245614	3.50E+06		8.30E+06	8.30E+06	2.49E+06	8.00E+05
**A0A0S2Z491**	Nucleophosmin	9	9	34.69387755	7.70E+06	4.35E+06	1.07E+07	1.69E+07	2.34E+07	2.40E+07
**A0A0U1RRM4**	Polypyrimidine tract-binding protein 1	17	17	42.17687075	3.70E+06	6.17E+06	7.40E+06	1.69E+07	1.38E+07	2.53E+07
**A0A140VJE8**	AP complex subunit beta	2	2	4.942166141					3.06E+06	2.60E+05
**A0A140VJJ2**	S-formylglutathione hydrolase	5	3	39.71631206				7.15E+06		8.50E+06
**A0A140VJS9**	Serine/threonine-protein phosphatase	8	8	25.80645161	4.90E+06	2.80E+06	1.50E+06	2.00E+07		1.30E+07
**A0A140VK93**	Adenylate kinase 2, mitochondrial	3	3	20.50209205	2.20E+06			3.83E+06	6.10E+06	1.10E+07
**A0A1B0GW77**	Alpha-aminoadipic semialdehyde dehydrogenase	5	5	12.45487365	4.05E+06		4.80E+06	4.80E+06	6.20E+06	
**A0A1C7CYX9**	Dihydropyrimidinase-related protein 2	8	4	17.42983752		1.10E+06	8.30E+06	1.30E+07	3.50E+06	5.75E+06
**A0A1S5UZ07**	Talin-1	4	4	2.697419859			1.00E+07	1.00E+07	2.40E+06	
**A0A1W2PQS6**	RPS10-NUDT3 readthrough	3	3	8.591065292	7.90E+06	5.50E+06	1.20E+06	3.50E+06	5.00E+06	2.40E+07
**A0A2R8Y891**	ATP-dependent 6-phosphofructokinase	5	2	5.889014723					5.90E+06	
**A0A2R8YD14**	40S ribosomal protein S24	3	3	14.54545455		1.30E+06		1.60E+07	3.80E+06	
**A0A2R8YEP4**	Flavin reductase (NADPH)	5	5	33.87755102	4.20E+06		4.10E+06	5.33E+06	1.20E+07	1.15E+07

**A0A2U3TZH3**	Elongation factor 1-alpha	20	9	46.97580645	1.99E+07	2.60E+06	1.90E+07	1.02E+07	1.68E+07	1.09E+07
**A0A2U3TZU2**	Glucose-6-phosphate isomerase	22	22	47.90619765	1.85E+07	9.07E+06	3.53E+07	5.05E+07	3.51E+07	2.33E+07
**A0A384MDR3**	Ferritin	4	4	32.57142857	3.60E+06	3.00E+06	4.10E+06	7.30E+06	7.40E+06	1.50E+07
**A0A384ME17**	Elongation factor Tu	9	9	21.75824176	4.80E+05	1.90E+06	6.90E+06	8.50E+06	1.00E+07	1.20E+07
**A0A384MTI8**	m7GpppX diphosphatase	2	2	8.605341246	1.10E+06			6.10E+06		9.70E+06
**A0A384N6C2**	Inosine-5'-monophosphate dehydrogenase	11	11	32.49027237	4.40E+06	1.90E+06	9.00E+06	1.02E+07	9.10E+06	5.60E+06
**A0A384NL00**	Glucose-6-phosphate 1-dehydrogenase	49	49	88.73786408	1.46E+07	1.70E+07	6.36E+07	7.27E+07	6.03E+07	6.03E+07
**A0A384NYT8**	Tubulin beta chain	22	5	65.61085973		2.75E+06	9.95E+06	1.50E+07	6.95E+06	1.56E+07
**A0A384P5L6**	Fumarylacetoacetase	3	3	9.785202864	3.00E+06				4.15E+06	
**A0A3B3ISG5**	Insulin-degrading enzyme	2	2	2.355250245	2.65E+06					
**A0A3B3ITK7**	Phosphoglucomutase-1	2	2	7.705479452						
**A0A3S6H7X4**	Protein arginine N-methyltransferase 1 transcript variant 5	2	2	5.735660848						
**A0A494C039**	Hypoxia up-regulated protein 1	9	9	14.5				3.10E+06	4.10E+06	2.80E+06
**A0A494C165**	Xaa-Pro dipeptidase	2	2	7.279693487	3.50E+06					2.55E+06
**A0A5F9ZHL1**	Acetyl-CoA acetyltransferase, mitochondrial	5	5	17.28110599	2.60E+06	1.80E+06		3.40E+06	6.20E+06	5.50E+06
**A2A274**	Aconitate hydratase, mitochondrial	3	3	7.080745342			5.60E+06	5.60E+06	3.20E+06	1.00E+07
**A2A3R6**	40S ribosomal protein S6	2	2	9.638554217						6.90E+06
**A3RJH1**	ATP-dependent RNA helicase DDX1	2	2	6.081081081				9.80E+05		
**A4D1P0**	Aldo_ket_red domain-containing protein	11	11	32.32044199	1.10E+07	2.90E+06	1.94E+07	3.96E+07	1.42E+07	7.63E+07
**A4D1U3**	Single-stranded DNA-binding protein, mitochondrial	7	7	52.02702703	5.50E+06		2.40E+06	9.70E+06	5.05E+06	1.85E+07
**A4D2P0**	Ras-related C3 botulinum toxin substrate 1 (Rho family, small GTP binding protein Rac1)	2	2	9.478672986			5.60E+06	4.10E+06	5.30E+06	1.50E+07

**A5PLM9**	Cathepsin L1	3	3	12.61261261			2.10E+06	2.30E+06	6.40E+06	2.30E+07
**A6NFX8**	ADP-sugar pyrophosphatase	4	4	20.68965517				4.00E+06		1.04E+07
**A8K2M0**	26S proteasome AAA-ATPase subunit RPT3	8	8	30.38277512			7.90E+06	4.85E+06	5.70E+06	
**A8K2U2**	Hexokinase	3	2	4.034896401	2.50E+06		4.60E+06	4.60E+06	5.60E+06	2.11E+06
**A8K3C3**	T-complex protein 1 subunit delta	8	7	20.03710575	4.85E+06	9.00E+06	5.95E+06	4.97E+06	1.23E+07	8.40E+06
**A8K401**	Prohibitin	8	8	48.16176471	1.60E+06	7.10E+05	3.70E+06	9.25E+06	7.90E+06	1.25E+07
**A8K486**	Peptidyl-prolyl cis-trans isomerase	13	12	59.39393939	4.17E+06	1.64E+07	1.71E+07	8.77E+07	3.71E+07	1.58E+08
**A8K4D5**	Kynureninase	6	6	22.15053763	1.75E+06		4.80E+06	3.95E+06	5.70E+06	6.15E+06
**A8K4W0**	40S ribosomal protein S3a	8	8	32.95454545			1.01E+07	7.27E+06	1.20E+07	1.88E+07
**A8K4Z4**	60S acidic ribosomal protein P0	10	10	38.80126183	9.90E+06	1.78E+06	5.10E+06	1.44E+07	6.30E+06	1.60E+07
**A8K7D9**	Importin subunit alpha	2	2	7.939508507	3.40E+06				4.50E+06	
**A8K8N7**	Formylglycinamide ribonucleotide amidotransferase	8	8	10.46337818	2.65E+06		5.50E+06	5.55E+06	6.05E+06	1.20E+06
**A8MXP9**	Matrin-3	2	2	4.469273743	3.90E+06	5.50E+06	5.60E+06	2.81E+07	1.84E+07	4.35E+07
**B0LPF3**	Adapter protein GRB2	2	2	16.58986175				1.10E+07	2.80E+06	
**B0QZ18**	Copine-1	2	2	4.612546125						2.20E+06
**B2R491**	40S ribosomal protein S4	20	13	55.89353612	2.60E+06		2.95E+06	1.14E+07	1.36E+07	2.07E+07
**B2R4C0**	60S ribosomal protein L18a	4	4	15.34090909			5.90E+06	3.85E+06	8.10E+06	1.05E+07
**B2R4R0**	Histone H4	15	15	59.22330097	1.43E+07	6.67E+07	6.55E+07	2.18E+08	1.24E+08	3.43E+08
**B2R5W3**	Poly [ADP-ribose] polymerase	7	7	12.72189349	2.80E+06		6.70E+06	6.70E+06	6.40E+06	
**P30085**	UMP-CMP kinase	2	2	9.210526316						
**B2R806**	Eukaryotic translation initiation factor 3 subunit E	3	3	7.415730337						
**B2RDD7**	Protein arginine N-methyltransferase 5	2	2	6.12244898	2.00E+06				3.90E+06	1.50E+06
**B3KSH1**	Eukaryotic translation initiation factor 3 subunit F	5	5	19.62365591			1.20E+07	1.20E+07	3.45E+06	3.70E+06
**B4DHQ3**	Phosphoserine aminotransferase	13	13	35.42168675	2.60E+06	1.30E+06	7.80E+06	5.70E+06	7.70E+06	6.75E+06

**B4DP80**	NAD(P)H-hydrate epimerase	6	6	27.03583062	4.30E+06	1.50E+06	6.60E+06	9.20E+06	8.40E+06	9.90E+06
**B4DR52**	Histone H2B	7	2	33.13253012			2.80E+06	1.68E+07	2.90E+07	6.30E+07
**B4DUC8**	S-methyl-5'-thioadenosine phosphorylase	3	3	19.33333333		8.50E+05				1.60E+07
**B5BUB1**	RuvB-like helicase	10	10	30.92105263	3.90E+06	1.70E+06	7.93E+06	1.03E+07	5.27E+06	5.13E+06
**B5BUB5**	Autoantigen La	9	9	28.67647059	6.00E+06	2.10E+06	9.50E+06	3.93E+06	5.73E+06	5.50E+06
**B7Z4C8**	60S ribosomal protein L31	5	5	30			7.30E+06	5.87E+06	6.20E+06	9.63E+06
**B7Z6Z4**	Myosin light polypeptide 6	3	3	15.96638655			3.50E+06	5.75E+06	3.30E+06	7.43E+06
**P49189**	4-trimethylaminobutyraldehyde dehydrogenase	4	4	9.459459459		6.10E+05				3.70E+06
**B9VP24**	60 kDa chaperonin	2	2	7.567567568		6.60E+05				
**C1PHA2**	Tyrosine-protein kinase receptor	2	2	2.8320971	2.20E+06					
**D3DPU2**	Adenylyl cyclase-associated protein	6	6	21.05263158		5.40E+06	5.20E+06	1.10E+07	9.50E+06	
**D3DUW5**	Dynamin-1-like protein	2	2	4.43599493				2.90E+05	3.30E+06	
**D6RBW1**	Eukaryotic translation initiation factor 4E	2	2	6.12244898				4.70E+06	8.30E+06	1.00E+07
**E1NZA1**	Peroxisome proliferator activated receptor interacting complex protein	5	5	43.31550802					3.50E+06	
**E5KMI6**	Lon protease homolog, mitochondrial	2	2	4.17101147		3.90E+05		9.40E+06		1.60E+06
**E5KNY5**	Leucine-rich PPR-motif containing	20	20	16.64275466	6.20E+06		7.45E+06	5.37E+06	7.52E+06	4.19E+06
**E5RJD8**	Tubulin-specific chaperone A	3	3	28.57142857			2.20E+06	1.10E+06		
**E7EPK1**	Septin-7	2	2	7.322654462				1.00E+07	1.60E+07	4.05E+06
**E7EQB2**	Lactotransferrin	2	2	3.017241379			1.50E+07	1.50E+07	5.98E+06	1.90E+06
**E9PCR7**	2-oxoglutarate dehydrogenase, mitochondrial	5	5	5.394990366			3.70E+06	3.70E+06		
**E9PK25**	Cofilin, non-muscle isoform	8	8	50.98039216	7.73E+06	1.22E+07	5.37E+06	3.62E+07	4.76E+07	4.25E+07
**E9PRY8**	Elongation factor 1-delta	4	4	8.177905308			2.80E+05	1.95E+06		9.75E+06
**F4ZW66**	NF110b	13	13	17.03786192	1.10E+06	6.80E+05	1.20E+07	8.00E+06	5.86E+06	4.07E+06

**F8W727**	60S ribosomal protein L32	3	3	15.68627451			3.00E+06	6.30E+06	9.40E+06	8.10E+06
**G4XXL9**	Cytochrome c	7	7	44.76190476	1.90E+06	2.15E+06	7.50E+06	1.06E+07	1.63E+07	2.30E+07
**G8JLB6**	Heterogeneous nuclear ribonucleoprotein H	9	7	29.23728814	7.70E+06	4.30E+06	4.28E+06	2.20E+06		1.12E+07
**G9K388**	YWHAE/FAM22A fusion protein	16	14	43.48958333	5.17E+06	8.67E+06	2.61E+07	2.13E+07	3.29E+07	2.77E+07
**H0Y8C6**	Importin-5	10	10	13.10282075	6.00E+06		1.40E+07	1.40E+07	6.45E+06	6.60E+06
**H0YFD6**	Enoyl-CoA hydratase	8	8	17.42424242	3.45E+06		1.10E+07	1.10E+07	8.10E+06	
**H0YKD8**	60S ribosomal protein L28	2	2	8.235294118			3.10E+06	2.93E+06	4.60E+06	6.90E+06
**H6VRG1**	Cytokeratin-1	38	33	51.9379845	5.20E+07	9.10E+06	7.19E+07	8.31E+07	4.82E+07	2.20E+07
**H7BY55**	Complement decay-accelerating factor	8	8	19.27272727		1.10E+06	7.45E+06	6.20E+06	7.53E+06	8.40E+05
**I3L0A0**	HCG2044781	3	3	4.594594595			1.10E+07	2.00E+07		9.47E+06
**I3L504**	Eukaryotic translation initiation factor 5A-1	9	9	37.09677419	3.45E+06	2.45E+06	7.45E+06	1.42E+07	2.00E+07	2.50E+07
**J3KPF3**	4F2 cell-surface antigen heavy chain	5	5	10.45958796	2.60E+06		1.30E+07	1.30E+07	8.20E+06	9.95E+05
**J3KQ18**	D-dopachrome decarboxylase	2	2	17.42424242	6.00E+06	2.00E+06			6.40E+06	1.40E+07
**J3KQ32**	Obg-like ATPase 1	9	9	30.28846154	6.55E+06	1.70E+06	2.00E+07	1.08E+07	6.40E+06	5.33E+06
**J3KQE5**	GTP-binding nuclear protein Ran	14	14	46.58119658	5.90E+06	5.85E+06	3.40E+07	2.10E+07	4.83E+07	5.37E+07
**J3KTA4**	DEAD box protein 5	7	4	12.70358306			6.70E+06	6.70E+06		2.70E+06
**J3QQ67**	60S ribosomal protein L18	7	7	32.10526316			9.55E+06	9.75E+06	7.00E+06	
**J3QQX2**	Rho GDP-dissociation inhibitor 1	6	6	36.59574468	1.80E+06	2.50E+06	9.20E+06	7.13E+06	2.00E+07	2.17E+07
**J9R021**	Eukaryotic translation initiation factor 3 subunit A	3	3	2.821997106	9.20E+05			1.95E+06	5.10E+06	1.04E+06
**K7ELC2**	40S ribosomal protein S15	5	5	53.94736842			1.20E+06	5.25E+06	9.90E+06	3.20E+06
**K7ER00**	Phenylalanine--tRNA ligase	2	2	3.832116788		1.40E+06		3.60E+06	5.80E+06	3.95E+06
**M0QYS1**	60S ribosomal protein L13a	5	5	20			1.80E+06	1.75E+06	7.60E+06	9.53E+06
**M0QYT0**	RRM domain-containing protein	4	4	13.08411215	3.20E+06		3.60E+06	6.60E+06	6.70E+06	1.33E+07
**M0R0R2**	40S ribosomal protein S5	6	6	28.44444444				2.87E+06		4.00E+06
**O00231**	26S proteasome non-ATPase regulatory subunit 11	11	11	27.48815166	3.20E+06	8.00E+05	6.80E+06	6.80E+06	9.40E+06	3.21E+06

**O00299**	Chloride intracellular channel protein 1	8	8	53.94190871	1.00E+06	2.70E+06		1.61E+07	4.20E+06	2.85E+07
**O00487**	26S proteasome non-ATPase regulatory subunit 14	2	2	10.64516129				4.40E+05	7.20E+06	5.30E+06
**O00625**	Pirin	2	2	7.24137931				4.90E+06	1.10E+07	1.10E+07
**O14980**	Exportin-1	7	7	9.337068161	2.10E+06	9.20E+05	7.80E+06	4.85E+06	3.70E+06	
**O15144**	Actin-related protein 2/3 complex subunit 2	5	5	18.66666667	2.30E+06				8.40E+06	6.90E+06
**O15460**	Prolyl 4-hydroxylase subunit alpha-2	2	2	5.420560748					2.00E+06	4.30E+05
**O43143**	Pre-mRNA-splicing factor ATP-dependent RNA helicase DHX15	9	9	15.47169811	1.90E+06	6.30E+06	7.60E+06	7.60E+06	4.80E+06	6.35E+06
**O43175**	D-3-phosphoglycerate dehydrogenase	9	9	22.32645403	3.10E+06	2.00E+06	8.20E+06	7.85E+06	6.10E+06	3.28E+06
**O43242**	26S proteasome non-ATPase regulatory subunit 3	5	5	11.23595506	6.20E+05					4.25E+06
**O43776**	Asparagine--tRNA ligase, cytoplasmic	7	7	13.50364964	1.30E+06	3.30E+06	4.70E+06	2.85E+06	1.50E+07	4.85E+06
**O60218**	Aldo-keto reductase family 1 member B10	28	28	83.5443038	1.02E+07	1.45E+07	3.42E+07	6.37E+07	5.38E+07	1.90E+08
**O60506**	Heterogeneous nuclear ribonucleoprotein Q	14	10	29.21348315	7.10E+06	4.37E+06	2.40E+07	2.05E+07	8.60E+06	2.10E+07
**O60701**	UDP-glucose 6-dehydrogenase	35	35	71.86234818	2.47E+07	6.43E+06	5.30E+07	5.22E+07	4.14E+07	2.77E+07
**O75367**	Core histone macro-H2A.1	8	8	32.52688172	3.20E+06	8.90E+05	5.20E+06	3.82E+06	1.20E+07	1.22E+07
**O75533**	Splicing factor 3B subunit 1	3	3	3.374233129	2.80E+06				3.70E+06	
**O75643**	U5 small nuclear ribonucleoprotein 200 kDa helicase	4	4	3.792134831	3.10E+06				4.70E+06	
**O75832**	26S proteasome non-ATPase regulatory subunit 10	2	2	6.637168142					7.10E+06	4.80E+06
**O76003**	Glutaredoxin-3	4	4	22.08955224				5.25E+06	5.10E+06	
**O76021**	Ribosomal L1 domain-containing protein 1	5	5	14.08163265	2.30E+06	1.45E+06	4.10E+06	8.40E+06	9.10E+06	1.07E+07
**O95373**	Importin-7	3	3	4.431599229	3.00E+06		3.40E+06	3.40E+06	5.00E+06	
**O95433**	Activator of 90 kDa heat shock protein ATPase homolog 1	2	2	7.396449704	3.30E+06	3.00E+06		2.56E+07	1.20E+06	2.80E+07

**P00338**	L-lactate dehydrogenase A chain	22	20	80.12048193	4.65E+06	5.85E+06	4.45E+07	5.20E+07	5.13E+07	1.53E+08
**P00492**	Hypoxanthine-guanine phosphoribosyltransferase	5	5	26.14678899	5.40E+06	1.40E+06	2.90E+06	1.80E+06	7.10E+06	1.10E+07
**P02533**	Keratin, type I cytoskeletal 14	11	3	23.94067797	3.70E+06					1.50E+06
**P04406**	Glyceraldehyde-3-phosphate dehydrogenase	25	25	83.58208955	5.13E+07	1.90E+07	1.63E+08	2.13E+08	2.22E+08	4.57E+08
**P04843**	Dolichyl-diphosphooligosaccharide--protein glycosyltransferase subunit 1	5	5	13.6738056	4.15E+06		8.20E+06	8.20E+06		
**P04844**	Dolichyl-diphosphooligosaccharide--protein glycosyltransferase subunit 2	2	2	6.814580032	1.95E+06				2.40E+06	
**P04908**	Histone H2A type 1-B/E	6	2	35.38461538			5.50E+05	2.05E+06	9.40E+05	
**P05120**	Plasminogen activator inhibitor 2	18	18	46.02409639	7.10E+06	3.80E+06	9.17E+06	1.60E+07	2.00E+07	1.45E+07
**P05198**	Eukaryotic translation initiation factor 2 subunit 1	8	8	31.42857143	9.70E+06		4.60E+06	8.10E+06	7.90E+06	1.33E+07
**P05787**	Keratin, type II cytoskeletal 8	25	18	53.00207039	4.10E+06	2.45E+06	1.69E+07	1.42E+07	1.68E+07	9.10E+06
**P06703**	Protein S100-A6	4	4	56.66666667			2.10E+06	5.03E+06	7.70E+06	1.67E+07
**P06733**	Alpha-enolase	45	40	84.33179724	1.37E+08	3.93E+07	2.78E+08	2.71E+08	3.30E+08	2.22E+08
**P06737**	Glycogen phosphorylase, liver form	29	23	40.25974026	4.95E+06		2.00E+07	1.29E+07	8.60E+06	7.10E+06
**P06899**	Histone H2B type 1-J	7	2	43.65079365	3.40E+06	3.20E+06	1.30E+07	5.20E+06		2.15E+07
**P07195**	L-lactate dehydrogenase B chain	21	19	66.46706587	6.90E+06	2.03E+06	3.05E+07	4.96E+07	5.68E+07	1.19E+08
**P07737**	Profilin-1	11	11	67.14285714	6.80E+06	6.15E+06	1.11E+07	3.57E+07	5.07E+07	5.23E+07
**P07741**	Adenine phosphoribosyltransferase	5	5	32.77777778	1.80E+06		5.70E+06	8.00E+06	1.10E+07	1.43E+07
**P07814**	Bifunctional glutamate/proline--tRNA ligase	9	9	7.076719577	1.80E+06	5.20E+05	4.80E+06	2.60E+06	3.35E+06	3.90E+06
**P07900**	Heat shock protein HSP 90-alpha	58	36	58.46994536	8.93E+06	8.80E+06	5.12E+07	5.73E+07	4.13E+07	5.67E+07
**P08238**	Heat shock protein HSP 90-	54	30	58.70165746	7.15E+06	5.15E+06	3.04E+07	3.23E+07	3.33E+07	3.97E+07

	beta									
**P08727**	Keratin, type I cytoskeletal 19	29	23	74.5	1.20E+06	3.05E+06	4.75E+06	7.70E+06	1.36E+07	1.33E+07
**P09382**	Galectin-1	4	4	35.55555556	2.00E+06			1.26E+07	7.39E+06	1.75E+07
**P09622**	Dihydrolipoyl dehydrogenase, mitochondrial	3	3	7.072691552					3.70E+06	3.35E+06
**P09960**	Leukotriene A-4 hydrolase	15	15	34.69721768	3.10E+06		8.70E+06	5.00E+06	4.70E+06	4.00E+06
**P0C0S5**	Histone H2A.Z	6	4	53.90625			6.40E+06	7.83E+06	5.06E+06	1.80E+07
**P10155**	60 kDa SS-A/Ro ribonucleoprotein	3	3	7.249070632	2.85E+06		5.00E+06	3.45E+06	6.60E+06	2.32E+06
**P10599**	Thioredoxin	4	4	42.85714286	1.30E+07	1.10E+06	1.23E+06	3.90E+07	2.68E+07	6.35E+07
**P11169**	Solute carrier family 2, facilitated glucose transporter member 3	3	2	5.443548387	3.90E+06		3.30E+06	3.30E+06		
**P11177**	Pyruvate dehydrogenase E1 component subunit beta, mitochondrial	2	2	5.292479109				6.40E+06	4.50E+06	6.60E+06
**P11216**	Glycogen phosphorylase, brain form	38	32	51.12692764	1.42E+07	4.40E+06	1.58E+07	1.21E+07	2.79E+07	7.20E+06
**P11940**	Polyadenylate-binding protein 1	12	7	22.16981132		2.30E+06	1.60E+07	9.00E+06	6.70E+06	9.13E+06
**P12004**	Proliferating cell nuclear antigen	3	3	15.70881226				7.00E+06	8.60E+06	6.80E+06
**P12081**	Histidine-tRNA ligase, cytoplasmic	9	9	23.37917485	4.30E+06	9.30E+05	1.30E+07	1.30E+07	3.08E+06	2.21E+06
**P12931**	Proto-oncogene tyrosine-protein kinase Src	3	3	4.664179104						
**P12956**	X-ray repair cross-complementing protein 6	20	20	44.99178982	4.95E+06	3.80E+06	9.70E+06	1.80E+07	1.02E+07	8.97E+06
**P13010**	X-ray repair cross-complementing protein 5	28	28	56.42076503	1.14E+07	4.40E+06	1.15E+07	1.14E+07	8.13E+06	8.37E+06
**P13489**	Ribonuclease inhibitor	7	7	21.90889371	2.57E+06	1.50E+06	7.70E+06	8.65E+06	5.69E+06	3.10E+06
**P13639**	Elongation factor 2	40	39	60.60606061	1.20E+07	1.13E+07	3.60E+07	3.64E+07	3.83E+07	3.26E+07
**P13645**	Keratin, type I cytoskeletal 10	32	28	59.24657534	2.88E+07	5.47E+06	2.88E+07	2.83E+07	2.26E+07	1.26E+07
**P13647**	Keratin, type II cytoskeletal 5	10	3	16.77966102	9.10E+05			3.60E+06	9.40E+06	
**P13674**	Prolyl 4-hydroxylase subunit	2	2	9.925093633			2.70E+06	3.50E+07	8.00E+06	

	alpha-1									
**P13797**	Plastin-3	13	10	29.68253968	2.10E+06	1.20E+06	1.00E+07	1.00E+07	4.03E+06	1.86E+06
**P14324**	Farnesyl pyrophosphate synthase	4	4	11.93317422						1.19E+07
**P14550**	Aldo-keto reductase family 1 member A1	6	5	22.76923077		2.50E+06	3.50E+06	4.10E+06	7.85E+06	8.30E+06
**P14868**	Aspartate-tRNA ligase, cytoplasmic	7	7	14.77045908			9.90E+06	9.90E+06	4.80E+06	5.90E+06
**P15121**	Aldo-keto reductase family 1 member B1	11	10	61.70886076			5.00E+06	9.00E+06	2.50E+07	2.43E+07
**P15311**	Ezrin	30	18	50.51194539	5.20E+06	9.80E+06	1.54E+07	1.80E+07	1.40E+07	6.15E+06
**P15559**	NAD(P)H dehydrogenase [quinone] 1	17	17	65.32846715	4.57E+06	2.90E+06	3.15E+07	2.56E+07	5.30E+07	1.30E+08
**P15880**	40S ribosomal protein S2	8	8	25.93856655			6.95E+06	1.05E+07	7.50E+06	5.87E+06
**P16152**	Carbonyl reductase [NADPH] 1	12	12	58.84476534	5.60E+06	1.10E+06	7.80E+06	1.96E+07	1.63E+07	3.40E+07
**P16402**	Histone H1.3	4	4	17.19457014			1.40E+07	1.62E+07	1.24E+07	2.90E+07
**P17174**	Aspartate aminotransferase, cytoplasmic	11	11	38.01452785	2.95E+06	1.50E+06	4.60E+06	5.60E+06	6.33E+06	1.12E+06
**P17931**	Galectin-3	6	6	28.4	5.65E+06	4.50E+06	3.15E+07	6.65E+07	4.68E+07	1.18E+08
**P17980**	26S proteasome regulatory subunit 6A	8	8	22.09567198		1.10E+06	6.90E+06	6.90E+06	4.13E+06	5.40E+06
**P17987**	T-complex protein 1 subunit alpha	8	8	25.89928058			1.40E+07	1.40E+07		3.90E+06
**P18077**	60S ribosomal protein L35a	2	2	7.272727273				6.10E+05		9.80E+06
**P18206**	Vinculin	11	11	12.43386243	5.80E+06		5.40E+06	9.40E+06	3.20E+06	1.44E+06
**P19338**	Nucleolin	24	24	29.01408451	6.85E+06	5.73E+06	2.83E+07	3.17E+07	2.22E+07	3.30E+07
**P19784**	Casein kinase II subunit alpha	2	2	5.142857143	7.90E+05		2.30E+06	4.90E+06		
**P20618**	Proteasome subunit beta type-1	7	7	34.02489627	1.02E+06		4.70E+06	5.80E+06	3.79E+06	8.13E+06
**P20700**	Lamin-B1	2	2	9.215017065						3.79E+06
**P21796**	Voltage-dependent anion-selective channel protein 1	12	11	53.71024735			5.85E+06	1.27E+07	1.10E+07	1.30E+07
**P22087**	rRNA 2'-O-methyltransferase fibrillarin	3	3	12.77258567				1.10E+07	8.80E+06	9.70E+06
**P22314**	Ubiquitin-like modifier-activating enzyme 1	40	40	53.02457467	1.01E+07	4.67E+06	2.23E+07	2.32E+07	2.91E+07	1.20E+07

**P22626**	Heterogeneous nuclear ribonucleoproteins A2/B1	17	13	40.50991501	1.00E+07	5.20E+06	9.83E+06	4.04E+07	2.10E+07	5.59E+07
**P23246**	Splicing factor, proline- and glutamine-rich	7	6	12.5884017	2.10E+06	1.65E+06	1.60E+06	3.47E+06	1.31E+07	1.40E+07
**P23284**	Peptidyl-prolyl cis-trans isomerase B	14	13	60.64814815	1.90E+07	4.80E+06	2.25E+07	2.66E+07	3.60E+07	5.50E+07
**P23368**	NAD-dependent malic enzyme, mitochondrial	3	3	9.931506849	2.60E+06	4.50E+05			9.70E+06	7.90E+05
**P23381**	Tryptophan--tRNA ligase, cytoplasmic	20	20	55.41401274	1.25E+07	4.15E+06	3.55E+07	4.79E+07	3.32E+07	1.91E+07
**P23396**	40S ribosomal protein S3	18	18	66.66666667	1.40E+06		1.30E+07	7.27E+06	1.17E+07	2.90E+07
**P23526**	Adenosylhomocysteinase	17	17	38.19444444	1.50E+07	2.00E+06	1.72E+07	2.20E+07	2.32E+07	1.33E+07
**P25398**	40S ribosomal protein S12	4	4	35.60606061			1.60E+07	8.47E+06	1.00E+07	1.93E+07
**P25788**	Proteasome subunit alpha type-3	6	6	26.2745098			5.30E+06	4.40E+06	1.50E+07	1.40E+07
**P25789**	Proteasome subunit alpha type-4	6	6	23.75478927	6.30E+06	2.50E+06	1.55E+07	1.04E+07	9.15E+06	2.73E+07
**P25815**	Protein S100-P	2	2	24.21052632						7.70E+06
**P26038**	Moesin	31	20	51.47313692	7.05E+06	5.17E+06	2.76E+07	2.51E+07	1.57E+07	1.88E+07
**P26196**	Probable ATP-dependent RNA helicase DDX6	2	2	6.211180124	4.20E+06					
**P26368**	Splicing factor U2AF 65 kDa subunit	4	4	7.157894737	5.20E+06		7.50E+06	7.50E+06		1.03E+07
**P26447**	Protein S100-A4	3	3	27.72277228	8.30E+06		2.60E+06	7.45E+06	7.48E+06	1.65E+07
**P26639**	Threonine--tRNA ligase 1, cytoplasmic	12	12	20.33195021	4.00E+06	4.70E+05	8.60E+06	3.95E+06	3.60E+06	2.75E+06
**P26641**	Elongation factor 1-gamma	15	15	36.61327231	8.47E+06	1.08E+07	7.40E+06	1.59E+07	1.16E+07	2.80E+07
**P27348**	14-3-3 protein theta	13	9	56.73469388	4.10E+06	5.27E+06	5.47E+06	5.73E+06	2.72E+06	2.95E+07
**P27635**	60S ribosomal protein L10	4	4	13.08411215			5.10E+06	5.20E+06	7.50E+06	9.90E+06
**P27695**	DNA-(apurinic or apyrimidinic site) endonuclease	5	5	23.27044025	9.10E+06	4.90E+06	7.30E+06	9.65E+06	2.01E+07	1.52E+07
**P27708**	CAD protein	4	3	1.887640449	3.30E+06				3.25E+06	1.90E+06
**P27824**	Calnexin	8	8	15.37162162	4.95E+06	5.63E+06	7.50E+06	1.00E+07	8.17E+06	5.60E+06
**P28074**	Proteasome subunit beta type-5	6	6	27.75665399		6.60E+05	4.10E+06			8.47E+06
**P28838**	Cytosol aminopeptidase	7	7	17.91907514	4.40E+06	7.00E+05			4.35E+06	1.30E+06

**P29401**	Transketolase	39	39	75.92295345	3.58E+07	8.73E+06	4.44E+07	5.54E+07	4.57E+07	5.00E+07
**P30040**	Endoplasmic reticulum resident protein 29	5	5	27.20306513	1.90E+06	2.70E+06		2.10E+07	6.70E+06	8.50E+06
**P30041**	Peroxiredoxin-6	20	20	79.01785714	4.11E+06	3.90E+06	1.27E+07	2.77E+07	2.78E+07	4.00E+07
**P30044**	Peroxiredoxin-5, mitochondrial	5	5	32.24299065	2.00E+06			6.90E+06	1.30E+07	7.80E+06
**P30048**	Thioredoxin-dependent peroxide reductase, mitochondrial	8	8	31.640625	7.25E+06	4.45E+06	3.90E+07	1.84E+07	1.09E+07	2.50E+07
**P30050**	60S ribosomal protein L12	3	3	24.24242424		8.60E+05	2.60E+07	1.90E+07	1.47E+07	4.57E+07
**P30101**	Protein disulfide-isomerase A3	35	29	64.15841584	3.00E+07	9.03E+06	5.10E+07	7.20E+07	4.63E+07	1.46E+07
**P30740**	Leukocyte elastase inhibitor	10	10	32.18997361	5.13E+06	1.80E+06	7.35E+06	7.50E+06	6.67E+06	3.55E+06
**P31930**	Cytochrome b-c1 complex subunit 1, mitochondrial	2	2	10.20833333			1.30E+07	1.30E+07		
**P31939**	Bifunctional purine biosynthesis protein ATIC	16	16	39.86486486	3.30E+06	2.20E+06	4.30E+07	1.27E+07	6.37E+06	3.84E+07
**P31947**	14-3-3 protein sigma	7	4	41.53225806	1.60E+06	1.40E+06	4.10E+06	8.95E+06	8.20E+06	2.94E+07
**P32929**	Cystathionine gamma-lyase	2	2	8.148148148	2.80E+06		5.40E+06	5.40E+06		
**P32969**	60S ribosomal protein L9	5	5	39.58333333			9.00E+05	7.75E+06	1.70E+07	1.34E+07
**P34897**	Serine hydroxymethyltransferase, mitochondrial	16	16	42.26190476	2.40E+06	1.35E+06	1.41E+07	9.38E+06	9.53E+06	1.77E+07
**P34931**	Heat shock 70 kDa protein 1-like	15	9	28.08112324	1.42E+07	3.95E+06	2.64E+07	3.50E+07	3.55E+07	1.63E+07
**P35080**	Profilin-2	2	2	15.71428571				1.90E+06		5.15E+06
**P35268**	60S ribosomal protein L22	4	3	30.46875				7.50E+06	2.10E+06	2.30E+07
**P35270**	Sepiapterin reductase	3	3	21.07279693	2.00E+06		1.80E+06	9.30E+05	7.90E+06	8.20E+06
**P35527**	Keratin, type I cytoskeletal 9	30	29	71.74959872	3.33E+07	2.34E+06	5.61E+07	8.10E+07	3.72E+07	1.96E+07
**P35606**	Coatomer subunit beta	3	3	5.298013245			4.20E+06	4.20E+06		
**P35908**	Keratin, type II cytoskeletal 2 epidermal	30	24	56.65101721	5.67E+06	4.34E+06	1.53E+07	1.10E+07	9.50E+06	5.53E+06
**P35998**	26S proteasome regulatory subunit 7	4	4	14.3187067	1.20E+06				3.90E+06	5.10E+06
**P36957**	Dihydrolipoyllysine-residue succinyltransferase component of 2-oxoglutarate	3	3	5.298013245				3.83E+06	3.70E+06	1.05E+07

	dehydrogenase complex, mitochondrial									
**P37802**	Transgelin-2	3	3	19.59798995	3.30E+06	2.20E+06		8.70E+06		7.10E+06
**P37837**	Transaldolase	15	15	39.16913947	3.64E+06	1.30E+06	1.19E+07	9.80E+06	1.83E+07	4.43E+07
**P38117**	Electron transfer flavoprotein subunit beta	7	7	26.66666667			4.20E+06	2.40E+06		9.20E+06
**P38159**	RNA-binding motif protein, X chromosome	4	4	10.99744246		1.20E+06	5.40E+06	5.07E+06		7.65E+06
**P38919**	Eukaryotic initiation factor 4A-III	9	6	24.57420925	2.60E+06	7.90E+06	5.70E+06	5.70E+06	5.20E+06	1.90E+06
**P39019**	40S ribosomal protein S19	6	6	30.34482759				5.80E+06	9.50E+06	9.80E+06
**P39023**	60S ribosomal protein L3	7	7	20.5955335			7.00E+06	5.35E+06	1.10E+07	1.31E+07
**P40925**	Malate dehydrogenase, cytoplasmic	7	7	28.44311377		1.20E+06	8.50E+06	6.15E+06	1.30E+07	2.23E+07
**P41091**	Eukaryotic translation initiation factor 2 subunit 3	10	10	30.93220339	4.10E+06		8.60E+06	8.60E+06	8.10E+06	6.90E+06
**P41250**	Glycine-tRNA ligase	20	20	38.15967524	5.65E+06	2.70E+06	9.70E+06	9.20E+06	1.02E+07	8.13E+06
**P46439**	Glutathione S-transferase Mu 5	3	2	15.13761468				1.55E+06		
**P46781**	40S ribosomal protein S9	14	14	37.62886598	4.05E+06	1.09E+06	1.27E+07	1.06E+07	1.90E+07	2.90E+07
**P46940**	Ras GTPase-activating-like protein IQGAP1	35	35	30.05431503	9.80E+06	4.00E+06	9.80E+06	1.19E+07	1.79E+07	9.43E+06
**P48163**	NADP-dependent malic enzyme	11	11	31.11888112	2.40E+06		2.10E+06	2.10E+06	3.88E+06	1.28E+06
**P48556**	26S proteasome non-ATPase regulatory subunit 8	4	4	11.71428571	8.30E+05			7.40E+06	5.90E+06	8.73E+06
**P49327**	Fatty acid synthase	67	67	37.71405814	1.38E+07	5.80E+06	1.88E+07	1.41E+07	1.83E+07	1.61E+07
**P49588**	Alanine-tRNA ligase, cytoplasmic	4	4	6.198347107			1.30E+07	1.30E+07	1.50E+06	2.00E+06
**P49721**	Proteasome subunit beta type-2	5	5	33.33333333	9.20E+06	7.30E+06	1.68E+06	2.83E+06	2.40E+06	
**P50213**	Isocitrate dehydrogenase [NAD] subunit alpha, mitochondrial	2	2	10.92896175				1.60E+07	1.40E+07	7.30E+06
**P50502**	Hsc70-interacting protein	3	3	8.401084011		1.20E+06	6.10E+06	6.10E+06	4.80E+06	
**P50990**	T-complex protein 1 subunit theta	9	9	21.71532847	1.10E+06	9.20E+05	5.20E+06	2.85E+06	9.00E+05	4.60E+06
**P51148**	Ras-related protein Rab-5C	3	3	20.37037037	7.00E+05			1.10E+07	1.80E+07	1.60E+07

**P51149**	Ras-related protein Rab-7a	2	2	11.5942029						5.90E+06
**P51572**	B-cell receptor-associated protein 31	2	2	7.317073171						5.80E+06
**P51991**	Heterogeneous nuclear ribonucleoprotein A3	9	7	23.54497354	6.00E+06		3.25E+06	1.50E+07	2.00E+07	1.54E+07
**P52209**	6-phosphogluconate dehydrogenase, decarboxylating	26	26	62.31884058	1.34E+07	7.40E+06	3.12E+07	2.90E+07	4.60E+07	3.63E+07
**P52597**	Heterogeneous nuclear ribonucleoprotein F	7	5	25.54216867	5.70E+06	1.00E+06	1.30E+07	4.04E+06		8.60E+06
**P52895**	Aldo-keto reductase family 1 member C2	24	4	80.18575851			1.20E+06	1.86E+07	2.15E+07	5.83E+07
**P53004**	Biliverdin reductase A	7	7	27.02702703	2.90E+06	1.00E+06		1.80E+06	3.20E+06	1.10E+07
**P53618**	Coatomer subunit beta	4	4	5.876180483	4.90E+06		7.80E+06	5.05E+06	2.80E+06	5.60E+06
**P53621**	Coatomer subunit alpha	7	7	8.088235294	4.30E+06	9.20E+05	3.35E+06	5.25E+06	3.75E+06	2.25E+06
**P53992**	Protein transport protein Sec24C	2	2	2.376599634	1.10E+06		4.10E+06	4.10E+06		
**P54136**	Arginine-tRNA ligase, cytoplasmic	11	11	18.18181818	3.30E+06		9.10E+06	9.10E+06	3.44E+06	1.97E+06
**P54886**	Delta-1-pyrroline-5-carboxylate synthase	7	7	11.82389937			3.40E+06	1.50E+07	3.50E+06	9.10E+06
**P55060**	Exportin-2	13	13	20.49433574	3.60E+06	2.80E+06	1.10E+07	1.10E+07	3.70E+06	2.85E+06
**P55072**	Transitional endoplasmic reticulum ATPase	30	30	48.63523573	1.10E+06	1.91E+06	3.16E+07	2.40E+07	1.04E+07	8.60E+06
**P55786**	Puromycin-sensitive aminopeptidase	16	16	21.54515778	5.95E+06	8.10E+05	1.30E+07	1.30E+07	7.25E+06	3.57E+06
**P56537**	Eukaryotic translation initiation factor 6	3	3	21.2244898	2.70E+06	4.20E+06		4.00E+06	1.70E+07	1.13E+07
**P60174**	Triosephosphate isomerase	20	20	74.82517483	2.69E+07	1.90E+07	9.93E+06	5.17E+07	2.15E+07	5.60E+07
**P60866**	40S ribosomal protein S20	2	2	19.32773109			6.10E+06	1.20E+07	1.20E+07	2.10E+07
**P60891**	Ribose-phosphate pyrophosphokinase 1	6	6	19.81132075		1.10E+06	3.11E+06	1.00E+07	1.00E+07	1.57E+07
**P61077**	Ubiquitin-conjugating enzyme E2 D3	5	5	42.85714286	1.70E+06		1.70E+06	6.75E+06		1.50E+07
**P61204**	ADP-ribosylation factor 3	7	5	52.48618785	5.50E+06	1.20E+06	1.60E+07	1.50E+07	1.60E+07	1.80E+07
**P61254**	60S ribosomal protein L26	3	3	12.4137931			3.80E+06	1.90E+06	5.50E+06	5.20E+06
**P61457**	Pterin-4-alpha-carbinolamine dehydratase	2	2	28.84615385						9.90E+06

**P61978**	Heterogeneous nuclear ribonucleoprotein K	11	11	33.90928726	1.44E+07	2.70E+06	8.80E+06	2.03E+07	1.56E+07	4.10E+07
**P61981**	14-3-3 protein gamma	15	12	56.68016194	5.30E+06	4.85E+06	9.70E+06	1.17E+07	7.25E+06	3.23E+07
**P62081**	40S ribosomal protein S7	6	6	38.1443299			5.90E+06	2.40E+06	1.60E+06	1.20E+07
**P62244**	40S ribosomal protein S15a	9	6	60.76923077			1.93E+07	1.11E+07	3.60E+07	3.10E+07
**P62249**	40S ribosomal protein S16	7	7	39.7260274	2.30E+06		8.85E+06	7.37E+06	7.70E+06	1.50E+07
**P62263**	40S ribosomal protein S14	3	3	15.23178808				3.70E+06	4.90E+06	
**P62269**	40S ribosomal protein S18	9	9	46.05263158			8.60E+06	1.06E+07	5.00E+06	1.70E+07
**P62277**	40S ribosomal protein S13	5	5	31.78807947	1.12E+07	1.31E+07	1.90E+07	2.40E+07	1.65E+07	4.53E+07
**P62280**	40S ribosomal protein S11	5	5	21.51898734			6.40E+06	3.15E+06	1.20E+07	1.10E+07
**P62314**	Small nuclear ribonucleoprotein Sm D1	3	3	36.97478992	6.45E+06	5.60E+06	1.26E+07	7.83E+06	1.11E+07	1.75E+07
**P62316**	Small nuclear ribonucleoprotein Sm D2	3	3	32.20338983				1.03E+07	3.00E+07	1.70E+07
**P62318**	Small nuclear ribonucleoprotein Sm D3	2	2	15.07936508	8.20E+06	7.00E+06	7.80E+06	2.87E+07	1.60E+06	4.10E+07
**P62424**	60S ribosomal protein L7a	10	10	37.96992481			6.50E+06	5.50E+06	1.80E+07	1.92E+07
**P62495**	Eukaryotic peptide chain release factor subunit 1	2	2	5.263157895				5.80E+06	5.80E+06	5.90E+06
**P62851**	40S ribosomal protein S25	5	5	29.6			2.20E+07	1.01E+07	1.38E+07	2.90E+07
**P62854**	40S ribosomal protein S26	3	3	31.30434783			6.50E+06	4.70E+06		2.00E+07
**P62888**	60S ribosomal protein L30	4	3	33.91304348	7.10E+05		1.20E+07	7.15E+06		6.10E+06
**P62906**	60S ribosomal protein L10a	6	6	27.64976959			6.10E+06	5.17E+06	1.50E+07	1.93E+07
**P62913**	60S ribosomal protein L11	3	3	16.85393258			2.20E+06	4.95E+06	5.00E+06	9.80E+06
**P62917**	60S ribosomal protein L8	6	6	20.62256809			2.00E+06	3.67E+06	3.60E+06	1.60E+07
**P62979**	Ubiquitin-40S ribosomal protein S27a	8	8	37.82051282	1.15E+07	7.30E+06	1.77E+07	5.57E+07	7.80E+07	7.56E+07
**P63104**	14-3-3 protein zeta/delta	17	13	73.46938776	5.10E+06	1.02E+07	8.90E+06	2.35E+07	8.90E+06	4.70E+07
**P63244**	Receptor of activated protein C kinase 1	13	13	61.829653	2.10E+06		4.15E+06	3.34E+07	9.10E+06	2.65E+07
**P67809**	Y-box-binding protein 1	3	3	16.04938272			5.60E+06			1.60E+07
**P68366**	Tubulin alpha-4A chain	18	3	47.32142857		3.10E+06		1.10E+06	4.30E+06	4.50E+06
**P68371**	Tubulin beta-4B chain	21	2	62.47191011	6.50E+05	1.10E+06	3.30E+06	3.20E+07		6.65E+06

**P78527**	DNA-dependent protein kinase catalytic subunit	19	19	5.789728682	6.10E+06		1.50E+07	1.50E+07	6.30E+06	2.84E+06
**P83731**	60S ribosomal protein L24	2	2	10.82802548				9.00E+06		
**P84098**	60S ribosomal protein L19	3	3	13.26530612				4.17E+06	1.00E+07	7.20E+06
**Q00688**	Peptidyl-prolyl cis-trans isomerase FKBP3	2	2	10.26785714						
**Q00796**	Sorbitol dehydrogenase	3	3	10.08403361					3.10E+06	5.20E+06
**Q00839**	Heterogeneous nuclear ribonucleoprotein U	12	12	20.48484848	1.10E+07	3.90E+06	1.61E+07	1.77E+07	6.68E+06	3.23E+07
**Q02790**	Peptidyl-prolyl cis-trans isomerase FKBP4	8	8	20.47930283	9.80E+06		4.60E+06	4.35E+06	7.40E+06	6.10E+06
**Q04695**	Keratin, type I cytoskeletal 17	7	2	13.19444444				1.80E+06	9.30E+06	1.11E+06
**Q04760**	Lactoylglutathione lyase	2	2	13.04347826			1.80E+06	2.70E+06		
**Q06210**	Glutamine-fructose-6-phosphate aminotransferase 1	2	2	3.147353362				6.30E+06	6.40E+06	7.40E+06
**Q06830**	Peroxiredoxin-1	22	2	79.89949749			1.60E+07	1.84E+07	1.40E+07	2.00E+07
**Q08211**	ATP-dependent RNA helicase A	20	20	22.51968504	2.50E+06	4.43E+06	1.30E+07	1.30E+07	1.00E+07	1.18E+07
**Q08J23**	RNA cytosine C(5)-methyltransferase NSUN2	2	2	3.780964798	3.90E+06					
**Q12797**	Aspartyl/asparaginyl beta-hydroxylase	2	2	3.693931398			4.70E+06	4.70E+06		
**Q12905**	Interleukin enhancer-binding factor 2	12	12	46.15384615	7.60E+06	2.83E+06	7.80E+06	8.77E+06	1.40E+07	1.32E+07
**Q12931**	Heat shock protein 75 kDa, mitochondrial	13	12	19.74431818	3.20E+06	6.20E+06	1.04E+07	1.06E+07	6.33E+07	8.17E+06
**Q13011**	Delta(3,5)-Delta(2,4)-dienoyl-CoA isomerase, mitochondrial	6	6	23.47560976	3.70E+05	1.80E+06		2.40E+06	7.30E+06	1.10E+07
**Q13228**	Methanethiol oxidase	4	4	10.80508475	3.20E+06				4.00E+06	
**Q13813**	Spectrin alpha chain, non-erythrocytic 1	57	57	30.17799353	4.77E+06	1.50E+06	1.03E+07	8.00E+06	9.80E+06	4.93E+06
**Q13838**	Spliceosome RNA helicase DDX39B	12	2	33.87850467	1.60E+06	2.20E+06			1.70E+06	4.30E+06
**Q13867**	Bleomycin hydrolase	2	2	6.813186813					1.50E+06	
**Q14103**	Heterogeneous nuclear ribonucleoprotein D0	10	9	25.35211268	5.80E+06	2.65E+06	1.00E+07	1.77E+07	1.62E+07	1.72E+07
**Q14204**	Cytoplasmic dynein 1 heavy	33	33	9.470512269	4.85E+06	2.49E+06	6.39E+06	5.63E+06	9.25E+06	5.70E+06

	chain 1									
**Q14315**	Filamin-C	7	3	2.165137615	9.80E+05				3.07E+06	4.00E+06
**Q15008**	26S proteasome non-ATPase regulatory subunit 6	7	7	21.85089974	1.30E+07		4.60E+06	4.60E+06	5.70E+06	
**Q15046**	Lysine-tRNA ligase	3	3	6.365159129					3.00E+06	
**Q15056**	Eukaryotic translation initiation factor 4H	3	3	17.74193548	3.00E+06			3.20E+06	1.90E+07	6.45E+06
**Q15149**	Plectin	6	6	1.750640478	4.90E+05				1.06E+06	
**Q15436**	Protein transport protein Sec23A	3	3	5.751633987	6.90E+05	6.90E+05	1.10E+07	1.10E+07	7.20E+06	7.80E+06
**Q15631**	Translin	3	3	20.61403509	1.40E+06			8.60E+06	1.50E+07	1.10E+07
**Q15717**	ELAV-like protein 1	2	2	7.36196319				1.50E+06	1.20E+07	1.40E+07
**Q16531**	DNA damage-binding protein 1	2	2	1.929824561	3.20E+06					
**Q16629**	Serine/arginine-rich splicing factor 7	3	3	19.74789916				2.40E+06		1.20E+07
**Q16658**	Fascin	12	12	32.45436105	6.90E+06	1.90E+06	8.25E+06	9.50E+06	5.83E+06	7.17E+06
**Q16881**	Thioredoxin reductase 1, cytoplasmic	21	21	48.53620955	2.07E+07	1.78E+06	3.05E+07	3.01E+07	1.67E+07	8.60E+06
**Q2XPP3**	Type II 3a-hydroxysteroid dehydrogenase variant	20	7	56.65634675	5.80E+06	1.45E+06	1.00E+07	1.25E+07	2.27E+07	2.80E+07
**Q32Q12**	Nucleoside diphosphate kinase	15	6	72.60273973	1.20E+07	3.90E+06	7.10E+06	1.12E+07	1.69E+07	4.30E+07
**Q3BDU5**	Prelamin-A/C	24	2	53.18275154			9.70E+06	2.40E+07	1.30E+07	1.04E+07
**Q4LE36**	ATP-citrate (pro-S-)-lyase	24	24	25.85751979	3.80E+06	9.55E+05	8.80E+06	9.00E+06	7.78E+06	4.30E+06
**Q4LE58**	eIF4G1 variant protein	6	6	3.81773399	1.60E+06	2.60E+06	1.90E+06	4.69E+06		5.20E+06
**Q53EU7**	N-myc downstream-regulated gene 1 protein	2	2	5.583756345					5.60E+06	
**Q53F64**	Heterogeneous nuclear ribonucleoprotein AB isoform a variant	6	5	22.89156627	4.35E+06	4.80E+06	6.00E+06	1.12E+07	1.72E+07	1.55E+07
**Q53FR4**	Vacuolar protein sorting 35 variant	8	8	12.18592965	3.95E+06		1.10E+07	1.10E+07	8.30E+06	4.00E+05
**Q53HB3**	Proteasome 26S ATPase subunit 1 variant	5	5	12.72727273	3.90E+06	1.30E+06	7.50E+06	5.45E+06	5.35E+06	5.40E+05
**Q53HV2**	T-complex protein 1 subunit eta	15	15	35.54327808	2.50E+06			3.90E+06	3.95E+06	3.70E+06

**Q562R1**	Beta-actin-like protein 2	13	2	28.19148936	1.30E+07			1.00E+07	9.20E+08	1.50E+06
**Q59EA2**	Coronin	3	3	6.786427146		5.30E+05				
**Q59EF6**	Calcium-activated neutral proteinase 2	2	2	3.155006859	2.20E+06		5.70E+06	3.21E+06		5.80E+06
**Q59EG8**	26S proteasome non-ATPase regulatory subunit 2	8	8	12.1577218	1.55E+06	3.30E+06	5.50E+06	4.00E+06	4.00E+06	5.00E+06
**Q59EJ5**	Glutathione S-transferase	9	8	46.32034632	2.75E+06	1.20E+06		2.77E+06	1.60E+07	1.09E+07
**Q59ER5**	WD repeat-containing protein 1 isoform 1 variant	6	6	10.8974359			3.20E+06	4.80E+06	5.80E+06	1.50E+06
**Q59FF0**	100 kDa coactivator	15	15	22.19917012		5.10E+05	8.30E+06	5.10E+06	1.20E+07	1.57E+06
**Q59FI4**	Importin 4 variant	7	7	9.335443038	3.20E+06				3.00E+06	6.80E+06
**Q59G24**	Activated RNA polymerase II transcriptional coactivator p15	2	2	12.68656716	1.40E+06		7.40E+06	1.40E+06		
**Q59G75**	Isoleucyl-tRNA synthetase	16	16	14.22986708	4.35E+06		5.67E+06	9.70E+06	6.07E+06	4.27E+06
**Q59GK9**	60S ribosomal protein L21	2	2	14.11042945				4.10E+06	2.00E+07	9.20E+06
**Q59GX9**	Ribosomal protein L5 variant	6	6	19.8019802		1.30E+06	1.04E+07	4.24E+06	4.60E+06	1.60E+07
**Q59GY2**	60S ribosomal protein L4	5	5	13.60544218				5.10E+07		1.80E+07
**Q59H77**	T-complex protein 1 subunit gamma	14	14	28.42287695	5.40E+06	6.20E+05	7.30E+06	8.85E+06	5.87E+06	5.80E+06
**Q59HH3**	Trifunctional purine biosynthetic protein adenosine-3	5	5	6.309751434		2.80E+06	3.10E+06	3.10E+06	2.40E+06	
**Q5JR94**	40S ribosomal protein S8	4	4	20.67307692			5.60E+05	3.20E+06	7.50E+06	6.60E+06
**Q5T5C7**	Seryl-tRNA synthetase	3	3	5.410447761			1.50E+06		3.20E+06	4.50E+06
**Q5VXV3**	SET	4	4	23.79310345	6.10E+06	6.30E+05		8.00E+06	9.91E+06	1.09E+07
**Q60FE5**	Filamin-A	54	48	29.84732824	3.60E+06	2.30E+06	8.00E+06	8.85E+06	1.03E+07	4.37E+06
**Q6FGH9**	Dynein light chain	3	3	14.60674157						5.85E+06
**Q6FHV6**	2-phospho-D-glycerate hydro-lyase	14	9	55.29953917	4.80E+06		7.00E+06	7.40E+06	5.90E+06	1.46E+06
**Q6FHX6**	Flap endonuclease 1	2	2	8.157894737				1.10E+07		3.30E+06
**Q6FI13**	Histone H2A type 2-A	9	5	57.69230769	8.17E+06	4.85E+06	1.14E+07	1.15E+07	1.75E+07	2.67E+07
**Q6FIC5**	Chloride intracellular channel protein	2	2	11.85770751				1.30E+06		7.20E+06

**Q6IAT9**	Proteasome subunit beta	6	6	43.09623431	4.21E+06	8.45E+06	5.55E+06	5.20E+06	9.68E+06	1.40E+07
**Q6NVY0**	Calcyclin-binding protein	4	4	33.33333333	2.10E+06			4.52E+06		1.60E+07
**Q6P2Q9**	Pre-mRNA-processing-splicing factor 8	3	3	1.541755889	4.20E+06		3.80E+06	3.80E+06	4.80E+06	3.10E+06
**Q6PCE3**	Glucose 1,6-bisphosphate synthase	3	3	5.466237942	2.10E+06		4.10E+06	4.10E+06	3.80E+06	
**Q6PKA6**	ALDH3A1 protein	21	20	44.3359375	2.73E+07	4.90E+06	6.43E+07	5.85E+07	4.84E+07	2.25E+07
**Q6V117**	MHC class I antigen	2	2	13.25966851		8.40E+05	7.70E+06	6.85E+06	1.20E+07	
**Q76LA1**	CSTB protein	2	2	24.48979592	2.10E+06			9.45E+06	1.80E+07	1.75E+07
**Q7RU04**	Aminopeptidase B	11	11	22.49240122	5.10E+06	1.20E+06	1.50E+07	1.02E+07	5.57E+06	3.45E+06
**Q7Z4X0**	MO25-like protein	2	2	5.571847507	1.90E+06	1.50E+06		4.90E+06		
**Q8N7G1**	Purine nucleoside phosphorylase	7	7	27.98634812	5.00E+06	4.40E+06		7.65E+06	1.20E+07	8.35E+06
**Q8NBS9**	Thioredoxin domain-containing protein 5	5	5	14.35185185	2.50E+06	2.80E+06	1.34E+07	1.48E+07	6.53E+06	2.20E+06
**Q8NBX0**	Saccharopine dehydrogenase-like oxidoreductase	4	4	18.18181818	1.80E+06	7.50E+05	6.60E+06	6.60E+06	4.80E+06	7.00E+05
**Q8TDN6**	Ribosome biogenesis protein BRX1 homolog	3	3	8.781869688		1.20E+06		7.40E+06	7.80E+06	6.55E+06
**Q92688**	Acidic leucine-rich nuclear phosphoprotein 32 family member B	10	5	29.88047809			2.70E+06	6.25E+06	9.60E+06	9.23E+06
**Q92973**	Transportin-1	11	11	15.47884187	3.45E+06		1.10E+07	1.90E+07	1.40E+07	1.05E+07
**Q969H8**	Myeloid-derived growth factor	3	3	17.34104046	6.30E+05		3.50E+06	2.00E+06	9.40E+06	1.20E+07
**Q96AG4**	Leucine-rich repeat-containing protein 59	4	4	16.61237785			9.10E+06	5.61E+06		3.70E+06
**Q96AZ6**	Interferon-stimulated gene 20 kDa protein	2	2	8.839779006	3.70E+06	3.90E+06		8.30E+06	1.30E+07	6.95E+06
**Q96G03**	Phosphoglucomutase-2	2	2	3.921568627	2.20E+06		5.90E+06	5.90E+06		
**Q96HE7**	ERO1-like protein alpha	15	15	37.60683761	6.70E+06	4.20E+06	1.17E+07	1.09E+07	1.13E+07	4.00E+06
**Q96KP4**	Cytosolic non-specific dipeptidase	6	6	18.94736842	9.50E+05	1.55E+06	5.90E+06	9.80E+06	4.20E+06	7.90E+05
**Q96P70**	Importin-9	2	2	4.41882805	4.60E+06				1.70E+06	
**Q96TA1**	Protein Niban 2	5	5	12.33243968	2.80E+06	1.50E+06	5.10E+06	8.30E+06	4.47E+06	1.08E+06

**Q99497**	Parkinson disease protein 7	11	11	65.60846561	1.80E+06	1.50E+06	1.10E+07	9.27E+06	1.10E+07	2.97E+07
**Q9BQG0**	Myb-binding protein 1A	3	3	3.012048193	1.50E+06					
**Q9BS26**	Endoplasmic reticulum resident protein 44	6	6	16.00985222			7.40E+06	7.40E+06	5.35E+06	
**Q9BTT0**	Acidic leucine-rich nuclear phosphoprotein 32 family member E	3	3	14.55223881	4.60E+06	4.60E+06	4.90E+06	2.10E+06		7.10E+06
**Q9BUF5**	Tubulin beta-6 chain	10	2	21.07623318				4.66E+06	8.40E+05	3.80E+06
**Q9BXP5**	Serrate RNA effector molecule homolog	3	3	3.082191781	2.15E+06		5.00E+06	5.00E+06	4.45E+06	
**Q9BYN0**	Sulfiredoxin-1	4	4	48.90510949		3.85E+06	8.90E+06	1.27E+07		2.20E+07
**Q9BZZ5**	Apoptosis inhibitor 5	4	4	8.58778626	1.50E+06		5.30E+06	3.55E+06	2.40E+06	
**Q9H2U2**	Inorganic pyrophosphatase 2, mitochondrial	3	2	8.982035928				3.50E+06	6.30E+06	7.15E+06
**Q9HAV7**	GrpE protein homolog 1, mitochondrial	3	3	20.2764977				6.40E+06		7.60E+06
**Q9HBB3**	60S ribosomal protein L6	9	9	33.56401384	5.10E+06	3.65E+06	8.40E+06	9.55E+06	1.30E+07	2.13E+07
**Q9HDC9**	Adipocyte plasma membrane-associated protein	2	2	3.846153846						
**Q9NQC3**	Reticulon-4	2	2	2.516778523	4.25E+06					
**Q9NR30**	Nucleolar RNA helicase 2	3	3	3.959131545				6.90E+06		5.20E+06
**Q9NR45**	Sialic acid synthase	3	3	11.69916435				9.00E+06	4.90E+06	1.00E+07
**Q9NSE4**	Isoleucine-tRNA ligase, mitochondrial	5	5	7.707509881		6.10E+05	6.80E+06	6.80E+06	4.70E+06	2.45E+06
**Q9NYU2**	UDP-glucose:glycoprotein glucosyltransferase 1	2	2	2.636655949					5.80E+06	
**Q9NZ23**	Drug-sensitive protein 1	6	6	57.35294118	5.20E+06	1.05E+06	4.43E+06	5.45E+06	7.63E+06	2.22E+06
**Q9NZM1**	Myoferlin	4	4	2.328966521		1.20E+06			3.50E+06	1.90E+06
**Q9UHG3**	Prenylcysteine oxidase 1	2	2	7.326732673	4.60E+06					
**Q9UMS4**	Pre-mRNA-processing factor 19	2	2	6.150793651	1.60E+06					
**Q9UNN8**	Endothelial protein C receptor	2	2	10.92436975		6.80E+05	7.00E+06	7.00E+06		5.80E+06
**Q9UQ80**	Proliferation-associated protein 2G4	13	13	46.0591133		1.40E+06	1.20E+07	7.55E+06	3.03E+06	6.33E+06
**Q9Y230**	RuvB-like 2	5	5	13.39092873			7.00E+06	6.45E+06	1.00E+07	1.00E+07

**Q9Y3U8**	60S ribosomal protein L36	2	2	21.9047619				8.80E+06		3.90E+06
**Q9Y5B9**	FACT complex subunit SPT16	2	2	3.533906399	2.20E+06					
**Q9Y678**	Coatomer subunit gamma-1	4	4	6.52173913	8.40E+05		4.50E+06	2.85E+06	3.13E+06	9.20E+06
**R4SBI6**	Epoxide hydrolase	16	16	45.27472527	1.02E+07	2.10E+06	1.25E+07	1.30E+07	1.14E+07	3.93E+06
**U3KQ56**	Glyoxylate reductase/hydroxypyruvate reductase	2	2	9.497206704			1.90E+06	1.70E+07	#DIV/0!	1.70E+07
**V9HW26**	ATP synthase subunit alpha	17	17	37.07052441	2.65E+06	1.30E+06	8.80E+06	1.02E+07	4.00E+06	1.02E+07
**V9HW31**	ATP synthase subunit beta	20	20	59.5463138	2.70E+06	3.80E+06	1.68E+07	1.34E+07	9.30E+06	1.47E+07
**V9HW55**	Proteasome subunit alpha type-1	12	12	46.8401487	1.80E+06	8.70E+06	1.10E+07	7.70E+06	2.10E+07	2.47E+07
**V9HW74**	Ubiquitin carboxyl-terminal hydrolase	17	17	82.95964126	5.45E+06	5.10E+06	1.70E+07	2.81E+07	2.12E+07	6.77E+07
**V9HW88**	Calreticulin	12	12	44.60431655	5.05E+06	2.20E+06	1.52E+07	1.47E+07	9.90E+06	8.97E+06
**V9HW90**	Glutathione reductase	10	10	32.95019157	2.20E+07	4.50E+06	1.47E+07	1.82E+07	1.84E+07	1.11E+07
**V9HW96**	CCT-beta	15	15	45.79439252	5.70E+06	1.59E+06	7.00E+06	9.30E+06	1.22E+07	1.14E+07
**V9HWB8**	Pyruvate kinase	44	42	77.58945386	1.08E+08	2.00E+07	9.27E+07	9.46E+07	8.03E+07	1.40E+08
**V9HWC9**	Superoxide dismutase [Cu-Zn]	2	2	32.46753247				2.00E+07		2.50E+07
**V9HWF4**	Phosphoglycerate kinase	44	44	89.20863309	6.51E+07	3.15E+07	2.25E+08	2.20E+08	2.38E+08	1.24E+08
**V9HWH9**	Protein S100	5	5	52.38095238	3.50E+06	8.20E+05	3.60E+06	5.87E+06		1.08E+07
**V9HWJ1**	Glutathione synthetase	8	8	16.4556962	4.65E+06	8.20E+05	5.90E+06	5.90E+06	7.32E+06	3.55E+06
**V9HWN7**	Fructose-bisphosphate aldolase	33	31	87.36263736	5.33E+07	2.00E+07	9.37E+07	1.63E+08	1.59E+08	1.62E+08
**X5DR03**	Glutathione transferase	3	2	18.34862385				1.40E+06		

Table S2. Proteins which expressions were significant changed between experimental keratinocyte groups (control group cultured with standard medium [**CTR**]; cell group irradiated by UVB (312 nm) at 60 mJ/cm^2 [**UVB**]; cell group cultured in medium containing 4μM CBD for 24h before and after UVB irradiation [**CBD+UVB+CBD**]; cell group cultured in medium containing 4μM CBD for 24h only after UVB irradiation [**UVB+CBD**]), their biological characteristics, fold-changes between cell groups (Empty cells in table used for "proteins were not found significantly changed") and their subcellular locations are indicated with a blue tick, they may come from the membranes associated with these locations in the membrane fractionation performed in this study.

			**Fold-changes**												
**Characteristic of proteins**	**protein ID**	**Protein name**	**UVB** vs. **CTR**	**CBD(*48h*)** vs. **CTR**	**CBD(*24h*)** vs. **CTR**	**CBD+UVB+CBD** vs. **UVB**	**UVB+CBD** vs. **UVB**	***cytosol***	***Plasma membrane***	***Extracellular exosome***	***Lysosome***	***Mitochondria***	***Golgi apparatus***	***Endoplasmic reticulum***	***Nucleus***
**Ribosomal Proteins**	P25398	40S ribosomal protein S12	68.65		129.73	1.18	2.28	✓					✓		✓
	P62244	40S ribosomal protein S15a	90.00		156.08	3.24	2.79	✓		✓					✓
	P62249	40S ribosomal protein S16	3.20	0.05	3.85	1.05	2.04	✓		✓					✓
	P62269	40S ribosomal protein S18	85.68		69.73	0.47	1.61	✓		✓					✓
	P39019	40S ribosomal protein S19	47.03			1.64	1.69	✓		✓					✓
	P62851	40S ribosomal protein S25	81.89		178.38	1.37	2.87	✓		✓					✓
	P23396	40S ribosomal protein S3	5.19	0.09	9.29	1.60	3.99	✓	✓	✓		✓		✓	✓
	A8K4W0	40S ribosomal protein S3a	58.92		81.49	1.65	2.59	✓							✓
	M0R0R2	40S ribosomal protein S5	23.24			0.04	1.40	✓							
	P62906	60S ribosomal protein L10a	41.89		49.46	2.90	3.74	✓		✓					✓
	P30050	60S ribosomal protein L12	153.78	6.97	210.81	0.77	2.41	✓		✓					✓
	M0QYS1	60S ribosomal protein L13a	14.16		14.59	4.35	5.46	✓							
	P84098	60S ribosomal protein L19	33.78			2.40	1.73	✓							✓
	P35268	60S ribosomal protein L22	60.81			0.28	3.07	✓		✓					✓

	H0YKD8	60S ribosomal protein L28	23.78		25.14	1.57	2.35	✓							
	B7Z4C8	60S ribosomal protein L31	47.57		59.19	1.06	1.64	✓							
	P62424	60S ribosomal protein L7a	44.59		52.70	3.27	3.49	✓							✓
	P62917	60S ribosomal protein L8	29.73		16.22	0.98	4.36	✓							
	Q59GX9	Ribosomal protein L5 variant	34.35	10.54	83.92	1.09	3.78	✓							
	Q4LE58	eIF4G1 variant protein	2.93	1.63	1.19	0.03	1.11	✓							
**Proteins participated in translation, proteasomal activity and protein ubiquitination**	I3L0A0	HCG2044781	162.16		89.19	0.01	0.47								✓
	A0A087X1Z3	Proteasome activator complex subunit 2	5.38	0.22	4.56	1.34	3.59	✓							✓
	P25788	Proteasome subunit alpha type-3	35.65		42.97	3.41	3.18	✓		✓					✓
	P49721	Proteasome subunit beta type-2	0.31	0.79	0.18	0.85	0.04	✓		✓					✓
	P28074	Proteasome subunit beta type-5		5.35	33.24		68.65	✓		✓					✓
	P50502	Hsc70-interacting protein	49.46	9.73	49.46	0.79	0.02	✓		✓					
	Q53HV2	T-complex protein 1 subunit eta	1.56	0.05	0.05	1.01	0.95	✓							
**Proteins with catalytic activity**	A0A5F9ZHL1	Acetyl-CoA acetyltransferase	1.31	0.69	0.05	1.82	1.62			✓		✓			
	P07741	Adenine phosphoribosyltransferase	4.44	0.07	3.17	1.38	1.79	✓		✓					✓
	A0A140VK93	Adenylate kinase 2	1.74	0.06	0.06	1.59	2.87					✓			
	P14550	Aldo-keto reductase family 1 member A1	33.24	20.27	28.38	1.91	2.02	✓	✓	✓					
	P15121	Aldo-keto reductase family 1 member B1	72.97		40.54	2.78	2.70	✓		✓	✓				✓
	O60218	Aldo-keto reductase family 1 member B10	6.26	1.42	3.36	0.85	2.98	✓			✓	✓			
	A0A0A0MSS8	Aldo-keto reductase family 1 member C3	2.91	0.78	0.12	1.91	2.51	✓		✓					✓
	P16152	Carbonyl reductase [NADPH] 1	3.50	0.20	1.39	0.83	1.73	✓		✓					
	P15559	NAD(P)H dehydrogenase [quinone] 1	5.61	0.64	6.90	2.07	5.08	✓							
	Q06830	Peroxiredoxin-1	148.92		129.73	0.76	1.09	✓		✓					✓
	V9HW26	ATP synthase subunit alpha	3.85	0.49	3.32	0.39						✓			
	P28838	Cytosol aminopeptidase	0.03	0.16	0.03	35.24	10.54	✓		✓			✓		✓

	Q96KP4	Cytosolic non-specific dipeptidase	10.32	1.63	6.21	0.43	0.08	✓		✓					✓
	Q13011	Delta (3,5)-Delta (2,4)-dienoyl-CoA isomerase	6.49	4.86	0.33	3.04	4.58	✓		✓		✓			
	P36957	Dihydrolipoyllysine-residue succinyltransferase component of 2-oxoglutarate dehydrogenase complex	31.08			0.97	2.73					✓			✓
	P14324	Farnesyl pyrophosphate synthase					96.22	✓							✓
	Q59G75	Isoleucyl-tRNA synthetase	2.23	0.03	1.30	0.63	0.44	✓				✓			
	B5BUB1	RuvB-like helicase	2.64	0.44	2.03	0.51	0.50								✓
	P37837	Transaldolase	2.69	0.36	3.25	1.87	4.52	✓							✓
	P55072	Transitional endoplasmic reticulum ATPase	21.79	1.73	28.68	0.44	0.36	✓		✓	✓			✓	✓
**Proteins participated in intracellular signaling**	O00299	Chloride intracellular channel protein 1	16.10	2.70	0.12	0.26	1.77	✓	✓	✓		✓			✓
	H7BY55	Complement decay-accelerating factor	50.27	8.92	60.41	1.22	0.14		✓						
	A0A024QZ77	EF-hand domain family, member D2	38.11		30.00	1.77	1.60	✓	✓						
	P38117	Electron transfer flavoprotein subunit beta	19.46		34.05	0.05	3.83					✓			
	A0A0S2Z3L0	Electron transfer flavoprotein subunit alpha	4.48	0.04	0.04	0.01	0.52					✓			
	Q99497	Parkinson disease protein 7	5.15	0.83	6.11	1.18	3.20	✓	✓	✓		✓			✓
	V9HWH9	Protein S100	1.68	0.23	1.03	0.02	1.84	✓	✓	✓					✓
	P06703	Protein S100-A6	40.81		17.03	1.53	3.31	✓	✓	✓					✓
	A4D2P0	Ras-related C3 botulinum toxin substrate 1	33.24		45.41	1.29	3.66	✓					✓		
	J3QQX2	Rho GDP-dissociation inhibitor 1	3.96	1.39	5.11	2.80	3.04	✓	✓						✓
**Proteins with DNA/RNA binding**	P52597	Heterogeneous nuclear ribonucleoprotein F	0.71	0.18	2.28	0.03	2.13	✓	✓						✓
	P38159	RNA-binding motif protein, X chromosome	41.08	9.73	43.78	0.02	1.51			✓					✓
	P62316	Small nuclear ribonucleoprotein Sm D2	83.70			2.91	1.65	✓		✓					✓
	P26368	Splicing factor U2AF 65 kDa subunit	1.44	0.02	1.44	0.02	1.38								✓
	P23246	Splicing factor, proline- and glutamine-rich	1.65	0.79	0.76	3.77	4.04	✓							✓

	G8JLB6	Heterogeneous nuclear ribonucleoprotein H	0.29	0.56	0.56	0.06	5.11	✓							✓
	A0A024RDR0	High-mobility group box 1	64.86		121.62	1.38	1.10								✓
	P16402	Histone H1.3	131.35		113.51	0.77	1.79								✓
	P0C0S5	Histone H2A.Z	63.51		51.89	0.65	2.30			✓					✓
	B4DR52	Histone H2B	136.49		22.70	1.72	3.74								✓
**Structural proteins**	A0A384NYT8	Tubulin beta chain	121.81	22.30	80.68	0.46	1.04	✓							
	Q9BUF5	Tubulin beta-6 chain	37.78			0.18	0.82	✓		✓					✓
	P08727	Keratin, type I cytoskeletal 19	6.42	2.54	3.96	1.77	1.73	✓	✓	✓					

Table S3. ID, name, function and average intensity list of proteins, which have adducts for malondialdehyde (**MDA**), in each experimental group: control group cultured with standart medium [CTR]; cell group cultured in medium containing 4 µM CBD for 48 h [CBD (48h)]; cell group cultured in medium containing 4 µM

CBD for 24h CBD [CBD (24h)]; cell group irradiated by UVB (312 nm) at 60 mJ/cm^2 [UVB]; cell group cultured in medium containing 4μM CBD for 24h before and after UVB irradiation [CBD+UVB+CBD]; cell group cultured in medium containing 4μM CBD for 24h only after UVB irradiation [UVB+CBD].

**Protein ID**	**Protein name**	**Function**	**CTR**	**CBD(*48h*)**	**CBD(*24h*)**	**UVB**	**CBD+UVB +CBD**	**UVB+CBD**
P31946	14-3-3 protein beta/alpha	cadherin binding, enzyme binding, MAPK cascade					1.90E+07	
B3GQS7	60 kDa chaperonin	protein refolding, isomerase activity		4.90E+06				4.00E+06
Q92688	Acidic leucine-rich nuclear phosphoprotein 32 family member B	activation of cysteine-type endopeptidase activity involved in apoptotic process (anti-apoptotic)					3.40E+04	
P12830	Cadherin-1	calcium-dependent cell adhesion	4.93E+06		1.10E+07			5.50E+06
P02458	Collagen alpha-1(II) chain	extracellular matrix structural constituent					6.00E+06	
Q9BTC0	Death-inducer obliterator 1	weakly pro-apoptotic when overexpressed, tumor suppressor	7.60E+07					
P63128	Endogenous retrovirus group K member 9 Pol protein	aspartic-type endopeptidase activity	7.12E+06					
P34931	Heat shock 70 kDa protein 1-like	protein folding, chaperone cofactor-dependent protein refolding			2.90E+07			

P14151	L-selectin	calcium-dependent cell-cell adhesion via plasma membrane cell adhesion molecules						1.10E+08
O00187	Mannan-binding lectin serine protease 2	calcium-dependent protein binding, serine-type endopeptidase activity	5.10E+07			3.80E+06		
P49790	Nuclear pore complex protein Nup153	protein-membrane adaptor activity, mRNA export from nucleus			2.10E+06			
A8K486	Peptidyl-prolyl cis-trans isomerase	protein folding			1.70E+07			5.20E+07
P21439	Phosphatidylcholine translocator ABCB4	phospholipid transporter activity, lipid metabolic process						6.75E+06
P35232	Prohibitin	proteinase activated receptor binding, regulation of apoptotic process, cell proliferation and NF-kappa-B signaling pathway			1.10E+07			
Q99623	Prohibitin-2	mitochondrial calcium ion transmembrane transport, regulation of apoptotic process and NF-kappa-B signaling pathway						2.55E+06
Q9GZR1	Sentrin-specific protease 6	SUMO-specific endopeptidase activity			3.90E+06			9.20E+06
A0A0U1RR07	Synaptotagmin-like protein 2	exocytosis , intracellular protein transport				4.70E+06		
Q15025	TNFAIP3-interacting protein 1	polyubiquitin modification-	5.08E+06					

		dependent protein binding, negative regulation of I-kappaB kinase/NF-kappaB signaling						
Q9UL52	Transmembrane protease serine 11E	serine-type endopeptidase activity					9.10E+06	
Q9BYE2	Transmembrane protease serine 13	serine-type endopeptidase activity						9.80E+05
Q9NRS4	Transmembrane protease serine 4	serine-type endopeptidase activity					4.20E+06	
A0A494C034	Tropomyosin alpha-3 chain	actin binding		4.80E+06				
Q6GMX0	Uncharacterized protein	no information				1.10E+08		
**Average**			**2.88E+07**	**4.85E+06**	**1.23E+07**	**3.95E+07**	**7.67E+06**	**2.39E+07**

Table S4. ID, name, function and average intensity list of proteins, which have adducts for 4-hydroxynonenal (**HNE**), in each experimental group: control group cultured with standart medium [CTR]; cell group cultured in medium containing 4 μM CBD for 48 h [CBD (48h)]; cell group cultured in medium containing 4 μM CBD for 24h CBD [CBD (24h)]; cell group irradiated by UVB (312 nm) at 60 mJ/cm^2 [UVB]; cell group cultured in medium containing 4μM CBD for 24h before and after UVB irradiation [CBD+UVB+CBD]; cell group cultured in medium containing 4μM CBD for 24h only after UVB irradiation [UVB+CBD].

Protein ID	Protein name	Function	CTR	CBD(*48h*)	CBD(*24h*)	UVB	CBD+UVB +CBD	UVB+CBD
P62081	40S ribosomal protein S7	regulation of intrinsic apoptotic signaling pathway by p53 class mediator						1.20E+07
A0A0C4DG17	40S ribosomal protein SA	structural constituent of ribosome					4.20E+06	
P18077	60S ribosomal protein L35a	cytoplasmic translation						7.20E+06
V9HWB4	78 kDa glucose-regulated protein	Misfolded protein binding, proteolysis involved in cellular protein catabolic process		4.40E+06		1.62E+07	6.25E+06	9.70E+06
O43423	Acidic leucine-rich nuclear phosphoprotein 32 family member C	Histone binding, regulation of apoptotic process				2.40E+07	2.40E+07	2.20E+07
A0A0S2Z3G9	Actinin alpha 4 isoform 1	Calcium ion binding regulation of apoptotic process				3.10E+06		
P06733	Alpha-enolase	negative regulation of hypoxia-induced intrinsic apoptotic signaling pathway and transcription by RNA polymerase				9.40E+07	3.00E+07	5.20E+07

A0A4D5RA7B7	Annexin	calcium-dependent phospholipid binding, calcium ion binding adaptive immune response				7.98E+06	4.00E+06	3.73E+07
P55289	Cadherin-12	calcium-dependent cell-cell adhesion via plasma membrane cell adhesion molecules, multicellular development				1.40E+07		
B4DNE1	cDNA FLJ52708, highly similar to Basigin	axon guidance, cell adhesion	3.80E+06					
A0A087WVQ6	Clathrin heavy chain	Protein kinase binding, intracellular protein transport	1.46E+07			6.80E+06	2.00E+07	
H0YKF0	Electron transfer flavoprotein subunit alpha, mitochondrial	electron transfer activity oxidoreductase activity,fatty acid beta-oxidation					1.80E+07	1.62E+07
Q16577	Elongation factor 1-alpha 1	translation elongation factor activity					1.50E+07	4.35E+07
A8K5I0	Epididymis secretory protein Li 103	ATP binding				1.15E+07	1.70E+07	
A0A384NPU5	Epididymis secretory sperm binding protein	protein disulfide isomerase activity	7.60E+06	9.35E+06		1.66E+07	5.05E+07	7.30E+07
P15311	Ezrin	protein localization to plasma membrane regulation of cell shape					5.90E+06	3.60E+06
B1AK88	F-actin-capping protein subunit beta	actin binding						1.60E+07

A0A024R321	Filamin B, beta (Actin binding protein 278)	Cell differentiation			1.00E+07	1.00E+07		
A0A0M4FNU3	Fructose-bisphosphate aldolase	fructose-bisphosphate aldolase activity	1.77E+07	6.20E+06		4.83E+07	7.95E+07	7.95E+07
A0A384NL00	Glucose-6-phosphate 1-dehydrogenase	negative regulation of ROS metabolic process, positive regulation of calcium ion transmembrane transport				8.00E+06	5.60E+06	
Q59EJ5	Glutathione S-transferase	glutathione transferase activity				8.40E+06		7.40E+06
P04406	Glyceraldehyde-3-phosphate dehydrogenase	aspartic-type endopeptidase inhibitor activity						9.50E+07
P07900	Heat shock protein HSP 90-alpha	chaperone-mediated autophagy, chaperone-mediated protein complex assembly, protein folding				5.00E+06		
P54652	Heat shock-related 70 kDa protein 2	Protein folding	1.25E+08	4.50E+07		2.40E+08	1.47E+08	8.20E+07
P51991	Heterogeneous nuclear ribonucleoprotein A3	mRNA binding, via spliceosome, RNA metabolic process				6.10E+06	2.90E+06	7.55E+06
P22626	Heterogeneous nuclear ribonucleoproteins A2/B1	mRNA processing						7.20E+06
V9HWB5	Inorganic diphosphatase	inorganic diphosphatase activity, magnesium ion binding	4.10E+06			2.40E+07	2.40E+07	2.45E+07

P13645	Keratin, type I cytoskeletal 10	keratinocyte differentiation				9.80E+06		
F5GZQ4	L-lactate dehydrogenase A chain	oxidoreductase activity, acting on the CH-OH group of donors, NAD or NADP as acceptor				1.30E+07	1.50E+07	1.20E+07
P26038	Moesin	positive regulation of cellular protein catabolic process regulation of cell shape and size					5.90E+06	3.60E+06
A0A024R1N1	Myosin, heavy polypeptide 9	actin filament binding	6.60E+06				1.19E+07	1.10E+07
F4ZW66	NF110b	DNA binding						1.00E+07
B5B2P4	Nuclear factor of activated T-cells c2 isoform IA-IIS-deltaXa	DNA binding transcription factor activity	1.70E+06					
Q02790	Peptidyl-prolyl cis-trans isomerase FKBP4	chaperone-mediated protein folding				1.11E+07	2.00E+07	1.00E+07
Q06830	Peroxiredoxin-1	removal of superoxide radicals, cell population proliferation,regulation of NIK/NF-kappaB signaling					1.10E+07	7.03E+07
A6NJJ0	Peroxiredoxin-4	antioxidant activity, oxidoreductase activity			7.90E+06	1.10E+07	1.80E+07	1.30E+07
P30044	Peroxiredoxin-5, mitochondrial	negative regulation of apoptotic process						8.45E+06

P30041	Peroxiredoxin-6	glutathione peroxidase activity,hydrogen peroxide catabolic process,cellular response to oxidative stress						7.60E+06
Q53G35	Phosphoglycerate mutase	bisphosphoglycerate mutase activity	2.50E+06			3.50E+06	1.90E+07	3.50E+07
A0A0S2Z489	Proteasome 26S subunit, non-ATPase, 12,	proteasome regulatory particle	2.30E+06					
Q53HB3	Proteasome 26S ATPase subunit 1 variant	protein catabolic process				5.50E+06		
A0A024RA52	proteasome subunit alpha type	ubiquitin-dependent protein catabolic process				1.10E+07		1.20E+07
A0A384NL22	Proteasome subunit beta	proteasome-mediated ubiquitin-dependent protein catabolic process					7.30E+06	5.00E+06
P30101	Protein disulfide-isomerase A3	positive regulation of extrinsic apoptotic signaling pathway, disulfide oxidoreductase activity	2.00E+07		5.80E+07	4.00E+07	8.60E+06	
Q6ZMV5	Protein PPP4R3C	metal ion binding, activation of protein kinase activity				1.30E+06		3.20E+07
Q9C0D5	protein TANC1	dendritic spine maintenance				7.40E+05		

V9HWB8	Pyruvate kinase	pyruvate kinase activity	1.07E+08			2.20E+08	2.10E+08	9.20E+07
Q9UL25	Ras-related protein Rab-21	intracellular protein transport						2.70E+06
O76021	Ribosomal L1 domain-containing protein 1	regulation of apoptotic process					8.30E+06	6.70E+06
A8K7F6	RNA helicase	RNA helicase activity, translation initiation factor activity				4.20E+06		
Q9HBR1	Sema domain-containing protein	semaphorin receptor binding	4.60E+06					
Q13813	Spectrin alpha chain, non-erythrocytic 1	calcium ion binding, endoplasmic reticulum to Golgi vesicle-mediated transport, MAPK cascade	3.45E+06				7.20E+06	
P23246	Splicing factor, proline- and glutamine-rich	RNA binding, RNA splicing, regulation of transcription	1.60E+06			3.31E+07	1.30E+07	4.50E+07
Q96SF2	T-complex protein 1 subunit theta-like 2	Protein folding, calcium-activated potassium channel activity unfolded protein binding					4.50E+06	
P55072	Transitional endoplasmic reticulum ATPase	Autophagy, cellular response to DNA damage stimulus ER-associated misfolded protein catabolic process, regulation of apoptotic process			3.25E+07	3.50E+07	1.85E+07	2.60E+07

Q6P2H8	Transmembrane protein 53	no information found	3.40E+06		4.30E+06	3.30E+06		
P23381	Tryptophan--tRNA ligase, cytoplasmic	negative regulation of cell population proliferation regulation of angiogenesis					6.00E+06	2.50E+06
F5H5D3	Tubulin alpha chain	microtubule-based process						1.90E+07
Q71U36	Tubulin alpha-1A chain	structural constituent of cytoskeleton, GTP binding, G2/M transition of mitotic cell cycle						1.90E+07
P68366	Tubulin alpha-4A chain	structural constituent of cytoskeleton,GTP binding,G2/M transition of mitotic cell cycle						1.90E+07
B2R6L0	Tubulin beta chain	GTP binding, structural constituent of cytoskeleton				1.23E+07	1.95E+07	4.81E+07
A0A384NYT8	Tubulin beta chain	GTP binding, structural constituent of cytoskeleton					2.50E+07	
J3KRJ1	Tumor protein p53-inducible protein 13	May act as a tumor suppressor. Inhibits tumor cell growth, when overexpressed			8.30E+06			3.97E+07
F4MHK7	Ubiquitously transcribed tetratricopeptide repeat protein Y-linked	histone demethylase activity (H3-K27 specific)			2.10E+06			

O60701	UDP-glucose 6-dehydrogenase	biosynthesis of glycosaminoglycans	7.30E+06				9.15E+06	
B2R6S5	UMP-CMP kinase	ATP binding, cytidylate kinase activity						3.60E+06
V9HWN7	Fructose-bisphosphate aldolase	fructose-bisphosphate aldolase activity	3.90E+06			2.50E+07	1.40E+07	3.35E+07
P12956	X-ray repair cross-complementing protein 6	double-strand break repair, telomere maintenance	4.00E+06		7.45E+06	9.73E+06	1.24E+07	6.95E+06
Q14624	Inter-alpha-trypsin inhibitor heavy chain H4	endopeptidase inhibitor activity	3.90E+07		1.60E+07			
Q15025	TNFAIP3-interacting protein 1	polyubiquitin modification-dependent protein binding, negative regulation of I-kappaB kinase/NF-kappaB signaling	2.15E+06		1.10E+06	1.80E+06	9.50E+05	1.00E+06
P48740	Mannan-binding lectin serine protease 1	calcium-dependent protein binding, serine-type endopeptidase activity	2.90E+06			1.60E+07	7.70E+06	1.20E+07
A0A5C2GKC1	IG c564	no information	2.50E+06					
P02765	Alpha-2-HS-glycoprotein	cysteine-type endopeptidase inhibitor activity	2.00E+06					
B2R4M6	Protein S100	Calcium ion binding	4.60E+06		3.50E+06			

O14514	Adhesion G protein-coupled receptor B1	G protein-coupled receptor activity, apoptotic cell clearance		2.00E+06				
Q86WV1	Src kinase-associated phosphoprotein 1	protein kinase binding, positive regulation of T-cell receptor signaling by enhancing the MAP kinase pathway				6.50E+06	2.30E+06	
P35232	Prohibitin	proteinase activated receptor binding, regulation of apoptotic process, cell proliferation and NF-kappa-B signaling pathway				3.00E+07		
Q14264	Endogenous retrovirus group 3 member 1 Env polyprotein	receptor recognition and membrane fusion during early infection						3.40E+06
Q9UMD9	Collagen alpha-1(XVII) chain	extracellular matrix structural constituen, epidermis development						1.10E+07
**Average**			**1.58E+07**	**1.34E+07**	**1.37E+07**	**2.69E+07**	**2.26E+07**	**2.43E+07**
